# Investigating One Health risks for human colonisation with extended spectrum β-lactamase-producing *Escherichia coli* and *Klebsiella pneumoniae* in Malawian households: a longitudinal cohort study

**DOI:** 10.1016/S2666-5247(23)00062-9

**Published:** 2023-07

**Authors:** Derek Cocker, Kondwani Chidziwisano, Madalitso Mphasa, Taonga Mwapasa, Joseph M Lewis, Barry Rowlingson, Melodie Sammarro, Winnie Bakali, Chifundo Salifu, Allan Zuza, Mary Charles, Tamandani Mandula, Victor Maiden, Stevie Amos, Shevin T Jacob, Henry Kajumbula, Lawrence Mugisha, David Musoke, Rachel Byrne, Thomas Edwards, Rebecca Lester, Nicola Elviss, Adam P Roberts, Andrew C Singer, Christopher Jewell, Tracy Morse, Nicholas A Feasey

**Affiliations:** a Malawi Liverpool Wellcome Research Programme, Kamuzu University of Health Sciences, Blantyre, Malawi; b Department of Clinical Sciences, Liverpool School of Tropical Medicine, Liverpool, UK; c Centre for Drugs and Diagnostics, Liverpool School of Tropical Medicine, Liverpool, UK; d Department of Tropical Disease Biology, Liverpool School of Tropical Medicine, Liverpool, UK; e Centre for Water, Sanitation, Health and Appropriate Technology Development, Malawi University of Business and Applied Sciences, Blantyre, Malawi; f Department of Civil and Environmental Engineering, University of Strathclyde, Glasgow, UK; g Department of Clinical Infection, Microbiology and Immunology, University of Liverpool, Liverpool, UK; h Centre for Health Informatics Computing and Statistics, Lancaster University, Lancaster, UK; i Global Health Security Department, Infectious Disease Institute, Makerere University, Kampala, Uganda; j Department of Medical Microbiology, Makerere University, Kampala, Uganda; k Department of Disease Control and Environmental Health, Makerere University, Kampala, Uganda; l College of Health Sciences, and College of Veterinary Medicine, Animal Resources and Biosecurity, Makerere University, Kampala, Uganda; m Conservation and Ecosystem Health Alliance, Kampala, Uganda; n Science Group, United Kingdom Health Security Agency, London, UK; o UK Centre for Ecology and Hydrology, Wallingford, UK

## Abstract

**Background:**

Low-income countries have high morbidity and mortality from drug-resistant infections, especially from enteric bacteria such as *Escherichia coli*. In these settings, sanitation infrastructure is of variable and often inadequate quality, creating risks of extended-spectrum β-lactamase (ESBL)-producing Enterobacterales transmission. We aimed to describe the prevalence, distribution, and risks of ESBL-producing Enterobacterales colonisation in sub-Saharan Africa using a One Health approach.

**Methods:**

Between April 29, 2019, and Dec 3, 2020, we recruited 300 households in Malawi for this longitudinal cohort study: 100 each in urban, peri-urban, and rural settings. All households underwent a baseline visit and 195 were selected for longitudinal follow-up, comprising up to three additional visits over a 6 month period. Data on human health, antibiotic usage, health-seeking behaviours, structural and behavioural environmental health practices, and animal husbandry were captured alongside human, animal, and environmental samples. Microbiological processing determined the presence of ESBL-producing *E coli* and *Klebsiella pneumoniae*, and hierarchical logistic regression was performed to evaluate the risks of human ESBL-producing Enterobacterales colonisation.

**Findings:**

A paucity of environmental health infrastructure and materials for safe sanitation was identified across all sites. A total of 11 975 samples were cultured, and ESBL-producing Enterobacterales were isolated from 1190 (41·8%) of 2845 samples of human stool, 290 (29·8%) of 973 samples of animal stool, 339 (66·2%) of 512 samples of river water, and 138 (46·0%) of 300 samples of drain water. Multivariable models illustrated that human ESBL-producing *E coli* colonisation was associated with the wet season (adjusted odds ratio 1·66, 95% credible interval 1·38–2·00), living in urban areas (2·01, 1·26–3·24), advanced age (1·14, 1·05–1·25), and living in households where animals were observed interacting with food (1·62, 1·17–2·28) or kept inside (1·58, 1·00–2·43). Human ESBL-producing *K pneumoniae* colonisation was associated with the wet season (2·12, 1·63–2·76).

**Interpretation:**

There are extremely high levels of ESBL-producing Enterobacterales colonisation in humans and animals and extensive contamination of the wider environment in southern Malawi. Urbanisation and seasonality are key risks for ESBL-producing Enterobacterales colonisation, probably reflecting environmental drivers. Without adequate efforts to improve environmental health, ESBL-producing Enterobacterales transmission is likely to persist in this setting.

**Funding:**

Medical Research Council, National Institute for Health and Care Research, and Wellcome Trust.

**Translation:**

For the Chichewa translation of the abstract see Supplementary Materials section.

## Introduction

Infections caused by antimicrobial-resistant (AMR) bacteria, especially extended-spectrum β-lactamase (ESBL)-producing Enterobacterales result in high morbidity and mortality in sub-Saharan Africa.^1^, ^2^ Given the heavy reliance on third-generation cephalosporins in human health, two of the most important AMR bacteria found in sub-Saharan Africa include *Escherichia coli*, responsible for a spectrum of community-acquired infections, and *Klebsiella pneumoniae*, typically associated with health-care-associated infection.^3^ These bacteria are present in the guts of humans and animals, and also within the broader environment.^4^ Households are therefore a focal point from which these enteric bacteria can disseminate via human and animal waste into the environment, potentially facilitating onward transmission of these bacteria to further human and animal hosts.^5^, ^6^

**Research in context**

**Evidence before this study**

Urban sites in sub-Saharan Africa are hotspots for antimicrobial resistance (AMR) due to environmental transmission. There are, however, few data describing and linking the prevalence and distribution of AMR-bacterial colonisation in humans, animals, and the environment. We searched PubMed on Sept 19, 2022, for studies in all languages, published from database inception to Sept 1, 2022, taking a One Health approach to exploring risk factors associated with human colonisation with extended-spectrum β-lactamase (ESBL)-producing bacteria in an African context. We used the search terms: ((ESBL) OR (Extended-spectrum beta-lactamase)) AND (((Angola OR Benin OR Botswana OR Burkina Faso OR Burundi OR Cameroon OR Cape Verde OR Central African Republic OR Chad OR Comoros OR Republic of the Congo OR Congo Brazzaville OR Democratic Republic of the Congo OR Cote d'Ivoire OR Djibouti OR Equatorial Guinea OR Eritrea OR Ethiopia OR Gabon OR The Gambia OR Ghana OR Guinea OR Guinea-Bissau OR Kenya OR Lesotho OR Liberia OR Madagascar OR Malawi OR Mali OR Mauritania OR Mauritius OR Mozambique OR Namibia OR Niger OR Nigeria OR Réunion OR Rwanda OR São Tomé and Principe OR Senegal OR Seychelles OR Sierra Leone OR Somalia OR South Africa OR Sudan OR Swaziland OR Eswatini OR Tanzania OR Togo OR Uganda OR Western Sahara OR Zambia OR Zimbabwe) OR Africa)). 17 studies were identified that evaluated environmental health or animal factors associated with human ESBL colonisation; however, these were frequently limited to hospital cohorts subject to selection bias or studies of suboptimal sample size. Nine studies identified relevant One Health factors associated with human ESBL colonisation, including use of unprotected or untreated drinking water, open defecation, shared toilets, inadequate handwashing, and in the case of two studies, a seasonal relationship to ESBL colonisation (adjusted odds ratios 2·21, 95% CI 1·07–8·75, and 2·9, 95% CI 1·3–5·6).

**Added value of this study**

This study provides longitudinal data from 300 households in urban, peri-urban, and rural Malawi, selected using a spatial design to enable unbiased estimates of community ESBL prevalence and assessment of regional variations. The microbiological sampling frame took a One Health approach, providing data from almost 12 000 samples, the results of which highlight extremely high levels of ESBL-producing Enterobacterales colonisation in humans and animals alongside extensive ESBL-producing Enterobacterales contamination of the environments in urban, peri-urban, and rural Malawi. Associations with human ESBL-producing Enterobacterales colonisation include animal exposures, household location (ie, urban *vs* rural), and the ability of the local environmental infrastructure to cope with increased rainfall.

**Implications of all the available evidence**

We demonstrate the key role of environmental health, as affected by seasonality, urbanisation, and animal cohabitation, on human carriage with ESBL-producing Enterobacterales bacteria in Malawi, adding to a growing body of evidence from sub-Saharan Africa highlighting the importance of adopting a One Health approach to future interventions to interrupt AMR transmission. Without adequate efforts to reduce ESBL-producing Enterobacterales contamination of the shared environment, through improved environmental hygiene at both a household and community level, we are unlikely to control the transmission of ESBL-producing Enterobacterales.

In low-income countries, paucity of infrastructure and services to support environmental health (including water, sanitation, food safety, and hygiene) is a key facilitator of unrestricted interaction between people and both human and animal waste in the environment. These infrastructural and service delivery inadequacies are compounded by poor hygiene practices, which increase the complexity of and opportunity for these interactions.^7^, ^8^ Environmental health factors are therefore thought to play a central role in environmental transmission of ESBL-producing Enterobacterales, which might lead to increased infection risks for vulnerable individuals.^7^ Interventions to interrupt community transmission of ESBL-producing Enterobacterales need to target key transmission routes, yet context-specific data to guide such interventions are lacking.

Transmission routes are likely to be heterogeneous across different settings; environmental health infrastructure and practices typically differ between urban and rural settings, with urban areas considered at particular risk of AMR transmission due to high-density housing, increased antibiotic use, and a paucity of environmental health infrastructure.^9^ Regional differences in animal ownership and husbandry practices are likely to further affect the risks of AMR transmission. Therefore, a One Health approach interrogating human, animal, and environmental health factors across urban and rural settings in low-income countries is essential for generating data to inform cost-effective interventions. To date, little evidence exists in the literature on the prevalence of ESBL-producing bacteria in African households and communities, especially One Health data incorporating contemporaneously collected data on the prevalence of ESBL colonisation in co-located animals and the local environment.^10^

Here, we have centred our study on households in urban, peri-urban, and rural settings in Malawi; our objectives were (1) to describe the prevalence of ESBL-producing Enterobacterales found in human, animal, and environmental compartments in southern Malawi, and (2) to identify key One Health factors associated with human ESBL-producing Enterobacterales colonisation to inform future interventions.

## Methods

### Study design and participants

The full methodological details of this longitudinal cohort study are available in a published study protocol and affiliated standard operating procedures online.^11^ Here, a summary of the information relevant to the scope of this study is presented.

We aimed to recruit a convenience sample of 300 households, 100 in each of Ndirande (urban), Chileka (peri-urban), and Chikwawa (rural), in the southern region of Malawi using GPS coordinates derived via an inhibitory with close pair spatial design to avoid systematic biases (as described by Cocker and colleagues^11^). This sampling strategy was necessitated by the absence of pre-existing data. Further details of the study sites are included in the study protocol and appendix 2 (p 2).^11^ Households identified at or near GPS locations were screened for inclusion and excluded if they (1) did not fall into study boundaries, (2) had fewer than two inhabitants, or (3) did not speak English or Chichewa. 65 households per region were assigned for longitudinal follow-up (four visits over 6 months) and the remaining 35 received only a baseline visit.

Ethical approval was obtained from the Liverpool School of Tropical Medicine Research and Ethics Committee, UK (18-090) and the College of Medicine Research and Ethics Committee, Malawi (P.11/18/2541). Permissions were granted by community leaders and support obtained from local community advisory groups. Sensitisations of study communities were conducted before study initiation, and informed written consent was obtained from all participants in their local language.

### Procedures

Case report forms were completed at each visit, providing information at both an individual and a household level on human health, antibiotic use, health-care seeking behaviour, structural and behavioural environmental health proxies, and animal husbandry. In parallel, observational checklists were completed, documenting key environmental health and household sanitation practices. Up to 20 human, animal, and environmental microbiological samples were taken per visit, inclusive of human and animal stool and a diverse range of environmental samples including: water (river water, household drinking water, and source water [ie, borehole or kiosk]), food (green leafy vegetables and fruit), participant contact samples (clothing and rinse water from hands), household environments (surfaces [ie, kitchen work surfaces or door handles] and household floors), and local drains (appendix 2 p 3).^11^ Samples were enriched in buffered peptone water at 37 °C (range 36–38) for 18–24 h, then inoculated onto CHROMagar ESBL chromogenic agar (CHROMagar, Paris, France) and incubated aerobically at 37°C (36–38) for 18–24 h, and read for growth of ESBL-producing bacteria (appendix 2 p 4).^11^ Colony colour and high-resolution melt-curve PCR techniques were used to speciate bacteria into ESBL-producing *E coli* or ESBL-producing *K pneumoniae.*^12^ Summaries of the sampling techniques, microbiological methods used, and quality assurance are available in appendix 2 (pp 3–4).

### Statistical analysis

Statistical analyses and graphic visualisations were performed using R version 4.1.2. Summaries are presented as proportions, medians (IQR), or means (SD). Kruskal-Wallis and Fisher's exact tests were used to test the equivalence of regional groups (ie, urban, peri-urban, and rural) for continuous and categorical variables, respectively. χ^2^ tests were used to determine associations between bacterial species composition of samples and seasonal variations in prevalence (wet [November–April] *vs* dry [May–October]). Principal component analysis (PCA) was used to visualise variation in the dataset across regions (urban, peri-urban, and rural) using the FactoMineR package in R.^13^ Putative individual-level variables (eg, age, sex), household-level variables (eg, household size, presence of toilet), and environmental contamination variables (eg, presence of ESBL-producing Enterobacterales in drain or stored water) likely to be associated with human ESBL-producing Enterobacterales colonisation were identified a priori by the DRUM consortium (appendix 2 pp 4–7), and PCA was performed on each group of variables after log-transforming continuous variables. Individuals and households were then plotted in PCA space for each of the groups of variables with 95% confidence ellipses for each region (ie, the region that contains 95% of all samples that can be drawn from the underlying normal distribution).

Logistic regression was used to identify factors associated with human ESBL-producing Enterobacterales colonisation; non-independence of within-participant and within-household samples was accounted for using hierarchical random effects. A variable selection strategy was used to construct the logistic regression models. The outcome variable was ESBL-producing Enterobacterales colonisation in human stool, with separate models fit for ESBL-producing *E coli* and ESBL-producing *K pneumoniae.* Individual and household variables were considered for inclusion. A stratified univariable analysis using logistic regression in each region separately was performed to determine which variables to include in the final analysis. Variables that were significantly associated with ESBL-producing Enterobacterales colonisation by univariable analysis (p<0·05) in any region were considered for inclusion in multivariable models, and those that were not significant or for which data were unavailable for at least one region were not included. Region and a random intercept per individual, which was nested within a random intercept per household, were included in the final models with the other selected variables. The models failed to converge when fit in a frequentist maximum likelihood framework, so they were fit using Bayesian logistic regression with Stan v2.21.0 via the R brms v2.13.5 package with default priors, four chains per dataset each with 2000 iterations in total, and 1000 warm-up iterations. Convergence of models was assessed by inspection of trace plots and by the closeness of the R-hat convergence diagnostic value to 1. Outputs were expressed as adjusted odds ratios (aORs) with 95% credible intervals (CrIs).

### Role of the funding source

The funders of the study had no role in study design, data collection, data analysis, data interpretation, or writing of the report.

## Results

**Figure 1:**
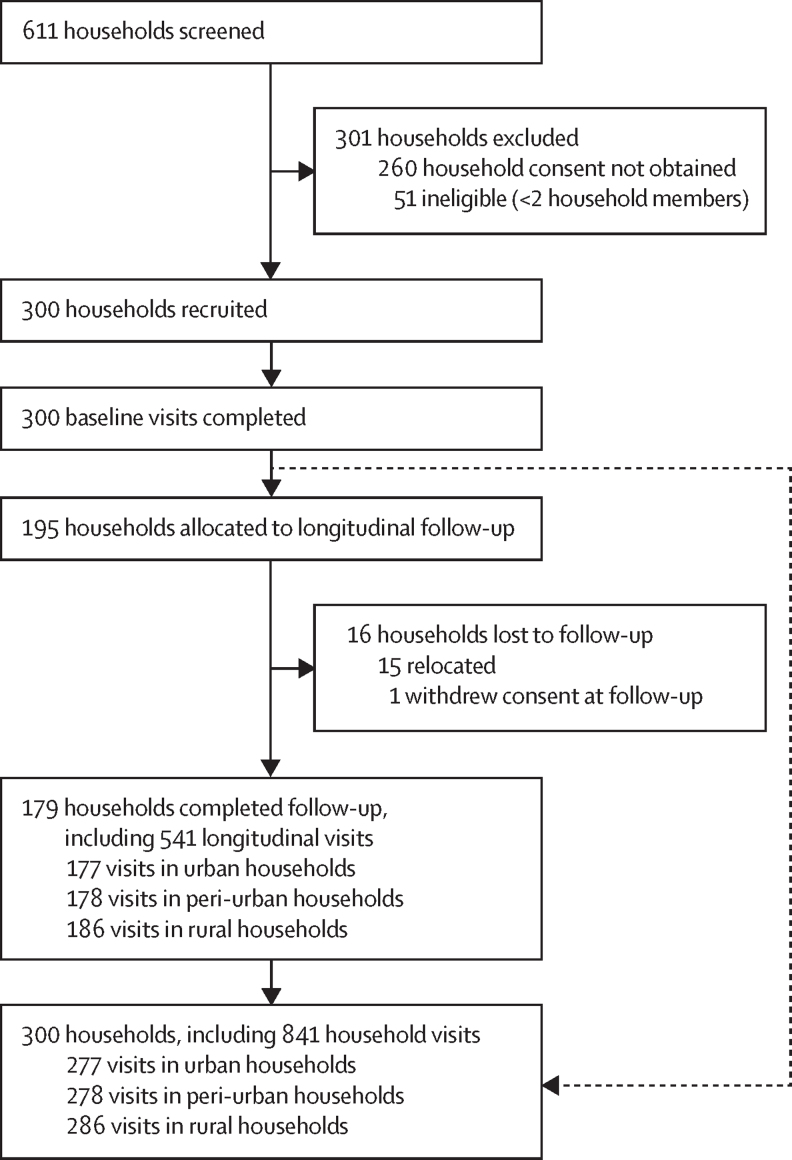
Households recruitment, visits, and loss to follow-up

Between April 29, 2019, and Dec 3, 2020, 611 households (263 urban, 229 peri-urban, and 119 rural) were screened and 300 households (100 per region) were recruited (figure 1). 179 households underwent longitudinal visits (four visits per house), 105 underwent a baseline visit only, and 16 households were lost to follow-up, providing a total of 841 visits. Across the 300 households, 965 (71·4%) of 1351 household members consented to recruitment.

The median number of residents per household was 4 (IQR 3–5; table). The median age of the study population was 18 years (7–34), and 545 (56·5%) of 965 household respondents were women (table). Although household income was higher in the urban and peri-urban regions than in the rural region, 293 (97·7%) of 300 households lived in absolute poverty, as defined by the World Bank (<US$1·90 per day per individual). HIV prevalence among the 473 residents with a reported HIV test was 66 (14·0%) and was highest in the peri-urban region, with high uptake of antiretroviral therapy (62 [93·9%]) and co-trimoxazole preventative therapy (60 [90·9%]).

**Table:** Baseline household and participant characteristics, stratified by region

		**Total**	**Urban**	**Peri-urban**	**Rural**	**p value**
**Household characteristics**						
Number of households		300	100	100	100	..
Number of household members		4 (3–5)	4 (3–5)	4 (3–6)	4 (3–5)	0·28
Household income (Malawi Kwacha/month)		30 000 (20 000–50 000)	50 000 (28 750–60 000)	40 000 (30 000–70 000)	20 000 (10 000–30 000)	<0·0001
**Individual characteristics**						
Age, years		18 (7–34)	15 (7–32)	20 (9–37)	17 (7–32)	0·031
Sex, male		420/965 (43·5%)	122/312 (39·1%)	170/383 (44·4%)	128/270 (47·4%)	0·12
Employment status						
	Student	372/965 (38·5%)	137/312 (43·9%)	140/383 (36·6%)	95/270 (35·2%)	0·059
	Unemployed	379/965 (39·3%)	107/312 (34·3%)	129/383 (33·7%)	143/270 (53·0%)	<0·0001
	Employed	214/965 (22·2%)	68/312 (21·8%)	114/383 (29·7%)	32/270 (11·8%)	<0·0001
**Health status and health-care exposures**						
Comorbidities, yes		66/965 (6·8%)	12/312 (4·0%)	24/383 (6·3%)	30/270 (11·1%)	0·0026
Living with HIV		66/965 (14·0%)	19/312 (10·5%)	26/383 (23·6%)	21/270 (11·3%)	0·026
Previous tuberculosis		12/965 (1·2%)	5/312 (2·0%)	4/383 (1·0%)	3/270 (1·1%)	0·76
Illness episode in last month		154/965 (16·0%)	35/312 (11·2%)	65/383 (17·0%)	54/270 (20·0%)	0·011
Health-care exposure as patient (last 6 months)		25/965 (2·6%)	6/312 (1·9%)	10/383 (2·6%)	9/270 (3·3%)	0·54
Health-care exposure as guardian (last 6 months)		28/965 (2·9%)	6/312 (1·9%)	8/383 (2·1%)	14/270 (5·2%)	0·046
Health-care exposure for work		4/965 (0·4%)	2/312 (0·6%)	1/383 (0·3%)	1/270 (0·4%)	0·83
Medication usage*						
Non-communicable disease medications		28/965 (2·9%)	12/312 (3·8%)	6/383 (1·6%)	10/270 (3·7%)	0·12
Antiretroviral therapy†		62/66 (93·9%)	17/19 (89·5%)	25/26 (96·2%)	20/21 (95·2%)	0·26
Co-trimoxazole preventative therapy†		60/66 (90·9%)	16/19 (84·2%)	24/26 (92·3%)	20/21 (95·2%)	0·11
**Antibiotic exposure in last 6 months**						
Antibiotic usage, yes		147/965 (15·2%)	51/312 (16·3%)	35/383 (9·1%)	61/270 (22·6%)	<0·0001

Data are median (IQR), n/N (%), or p value.

* Ongoing at baseline.

† Adjusted for individuals with HIV at each site.

Non-infectious comorbidities were infrequent, with 66 (6·8%) of 965 of the respondents reporting chronic conditions and only 28 (2·9%) taking any form of regular medication other than antiretroviral therapy or co-trimoxazole preventative therapy (table). There were low levels of recent health-care exposure, although 147 (15·2%) participants had received antibiotics in the previous 6 months (table), predominantly limited to oral amoxicillin (64 [35·4%]), co-trimoxazole (65 [35·9%]), and metronidazole (23 [12·7%]; appendix 2 p 11). Children were more likely to have been prescribed antibiotics in the previous 6 months, especially those younger than 5 years (appendix 2 p 12).

176 (58·7%) of 300 households reported cohabitation with domestic or livestock animals, with 36 (36·0%) of 100 urban, 59 (59·0%) of 100 peri-urban, and 81 (81·0%) of 100 rural households owning at least one animal (appendix 2 p 13). 2169 animals were linked to a study household at baseline, and both the composition of species and number of animals present per household varied by region (appendix 2 pp 13–15). Companion animals (ie, cats and dogs) were kept in low numbers per household and made up a large proportion of the animal species owned in urban (23 [63·9%] of 36) and peri-urban (25 [42·4%] of 59) households. Many households kept poultry (ie, chickens, doves, and ducks), with chickens both the most commonly owned and most numerous animals (18 [18·0%] of urban, 39 [39·0%] of peri-urban, and 59 [59·0%] of rural households owned chickens). Larger animals (ie, pigs, goats, and cattle) were seen at fewer households and primarily in the rural or peri-urban settings. 77 (25·7%) of 300 households specifically owned animals for breeding and selling purposes, especially in the rural area.

We observed regional differences in animal husbandry practices, with animals frequently kept inside the house in the urban setting, particularly poultry (appendix 2 pp 13–15). Co-located animals often had episodes of presumed illness, especially poultry (51 [44·3%] of 115 households), but households had limited access to, or awareness of, veterinarian services (47 [26·9%] of 175 households). Households reported that they would often do nothing if animals became unwell (appendix 2 p 15), and only seven (4·0%) of 176 households treated any animals with antibiotics before recruitment into the study (appendix 2 p 13).

Households typically obtained water from boreholes (153 [48·7%] of 300 households), public kiosks (79 [25·2%]), or taps piped into the household compound (53 [16·9%]; appendix 2 pp 16–17). Water was infrequently treated before drinking (25 [8·3%] of 300 households) and often left uncovered when stored (143 [31·9%] of 448 household visits). Access to infrastructure and consumables for adequate hand hygiene was limited, with only 123 (41·0%) households having a handwashing facility. 267 (89·0%) households owned a toilet, most commonly a pit latrine (137 [88·8%] of 267 households), and households frequently shared their toilet with other non-household members (112 [41·9%] of 267 households). Anal cleansing materials were identified at 133 (18·9%) of 703 toilet visits. Open defecation was common in households (86 [28·7%] of 300), and human faeces were often found on the floor in or around the household (66 [8·1%] of 814 visits; appendix 2 pp 16–17). Only 13 (4·3%) households had adequate management of animal faeces, and 24 (8·0%) had adequate waste management of household rubbish.

Households relied on local markets for purchasing vegetables (260 [86·7%] of 300) and frequently ate street food (267 [89·0%]). Cooked food was often seen to be covered (286 [92·3%] of 310 observations), but raw fruit and vegetables (131 [38·1%] of 344 observations) and cooking utensils (129 [15·9%] of 813 observations) were often left uncovered. Animals in households were often seen in contact with human food (123 [62·8%] of 196 observations) and were frequently present in food preparation areas (196 [24·1%] of 813 observations).

Key environmental exposures identified via direct observations or reported behaviours included contact by humans—particularly children—and animals with standing water (16 [24·6%] child–water interactions and 33 [50·8%] animal–water interactions in 65 observations), sewerage via open drains (28 [20·4%] child–drain interactions and 60 [43·8%] animal–drain interactions in 137 observations), and the local river network (66 [22·0%] child–river interactions and 99 [33·0%] adult–river interactions in 300 observations; appendix 2 pp 16–17).

**Figure 2:**
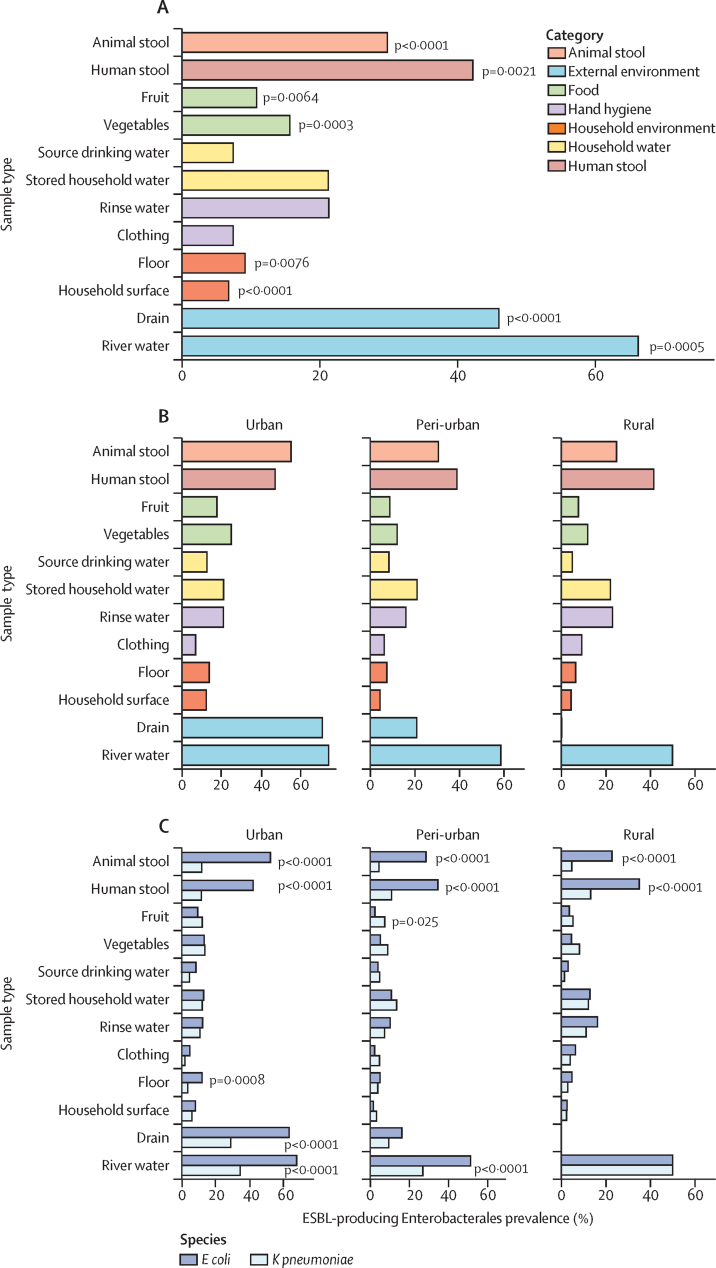
Regional differences in the prevalence of ESBL-producing Enterobacterales colonisation and environmental contamination (A) Proportion of samples positive for ESBL-producing Enterobacterales at urban, peri-urban, and rural households. p values indicate significant regional differences in the prevalence of ESBL-producing Enterobacterales for each sample type. (B) Breakdown of urban, peri-urban, and rural proportions of ESBL-producing Enterobacterales. (C) Proportion of the household human stool, animal stool, and environmental samples positive for ESBL-producing *Escherichia coli* or ESBL-producing *Klebsiella pneumoniae*, stratified by sample type, bacterial species, and region. Significant variations in the proportion of ESBL-producing *E coli vs* ESBL-producing *K pneumoniae* by sample type, assessed by χ^2^ test, are shown. ESBL=extended spectrum β-lactamase.

In total, 11 975 samples (2845 human stool, 973 animal stool, and 8157 environmental samples) were cultured, from which ESBL-producing *E coli* or ESBL-producing *K pneumoniae* were isolated from 1190 (41·8%) human stool samples and 290 (29·8%) animal stool samples (figure 2; appendix 2 p 18). Animal species with particularly high rates of ESBL-producing Enterobacterales colonisation included pigs (21 [56·8%] of 37), poultry (148 [32·5%] of 455), and dogs (30 [58·8%] of 51; appendix 2 p 28). ESBL-producing *E coli* or ESBL-producing *K pneumoniae* were also isolated from a range of household environment, hand hygiene, food, and community environment samples, with 339 (66·2%) of 512 river water samples and 138 (46·0%) of 300 drain samples containing ESBL-producing Enterobacterales.

Among the 195 households with longitudinal follow-up, 191 (97·9%) had at least one ESBL-producing Enterobacterales-colonised household member, and 50 (41·7%) of the 120 households that owned animals had at least one ESBL-producing Enterobacterales-colonised animal stool. EBSL-producing Enterobacterales-contaminated food was detected in 108 (55·4%) of 195 households, and 89 (45·6%) had ESBL-producing Enterobacterales-contaminated environments during the study. Longitudinal follow-up revealed a high degree of flux in ESBL-producing Enterobacterales status in human household members, with 588 (78·7%) of 747 individuals carrying ESBL-producing Enterobacterales at some point (appendix 2 pp 29–31).

Marked regional differences in the prevalence of ESBL-producing Enterobacterales colonisation and contamination were noted (figure 2). Higher rates of ESBL-producing Enterobacterales were found in the urban settings, inclusive of animal stool, human stool, food, household environment, and local drainage and river networks. The prevalence of ESBL-producing Enterobacterales was greater in the wet season than in the dry season in human stool (47·2% [SD 49·9] in the wet season *vs* 36·6% [48·2] in the dry season, p<0·0001), animal stool (33·3% [47·2] *vs* 25·5% [43·6], p=0·010), stored drinking water (26·2% [44·0] *vs* 15·2% [35·9], p<0·0001), household floors (11·5% [31·9] *vs* 6·6% [24·9], p=0·031), and household environments (8·8% [28·3] *vs* 4·5% [20·8], p<0·0001; appendix 2 p 18).

**Figure 3:**
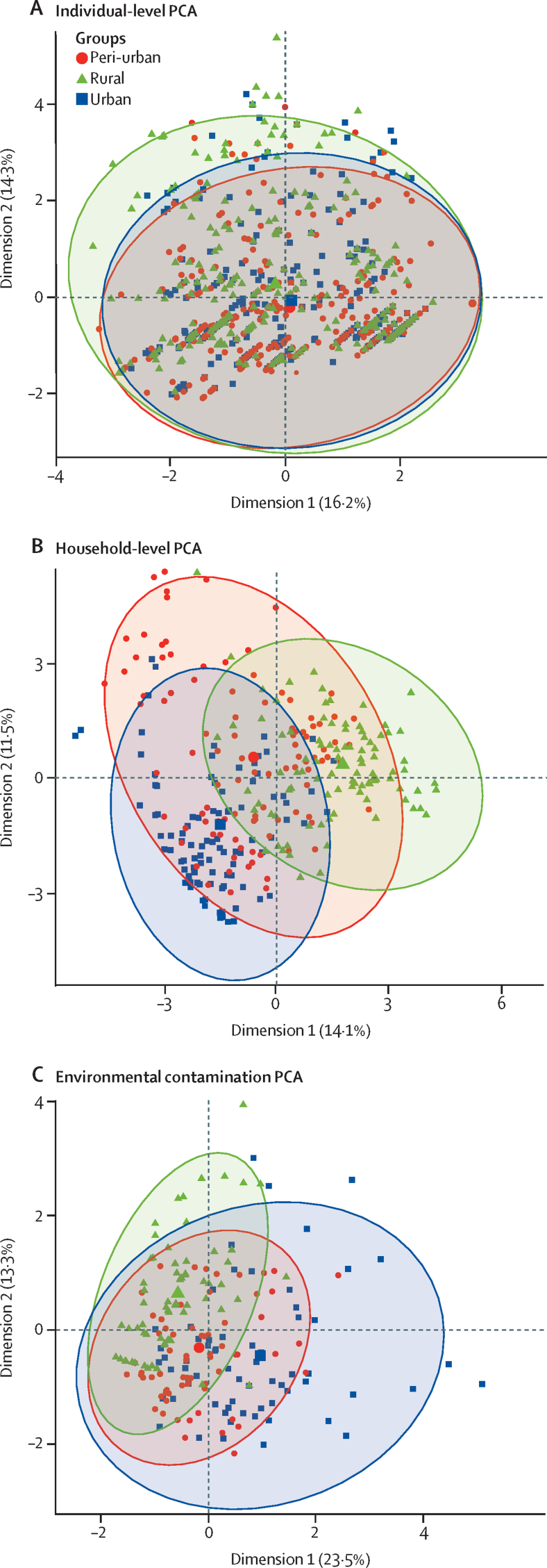
Confidence ellipses of regional effects for the (A) individual-level, (B) household-level, and (C) environmental contamination datasets, from the first two principal components Points in (A) represent individuals and points in (B) and (C) represent households. PCA=principal component analysis.

We used PCA to assess regional differences in individual-level, household-level, and environmental contamination variables (figure 3; appendix 2 pp 29–31). Projection of individuals or households onto PCA space, stratified by region, identified few differences at an individual level across regions (figure 3A), but identified regional differences in distributions of household-level (figure 3B) and environmental contamination (figure 3C) variables, consistent with differences in animal husbandry and environmental health behaviours and ESBL-producing Enterobacterales contamination across urban, peri-urban, and rural areas.

**Figure 4:**
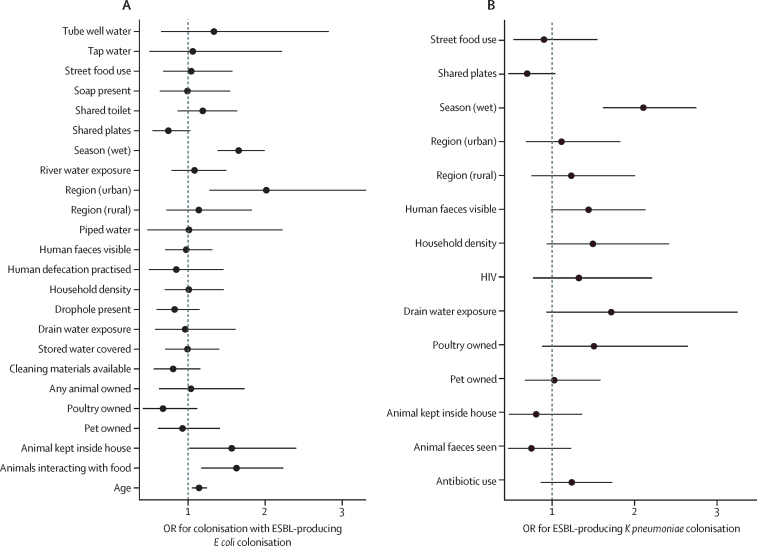
Parameter estimates for the fixed effects used in a multivariable logistic regression model of (A) ESBL-producing *Escherichia coli* and (B) ESBL-producing *Klebsiella pneumoniae* colonisation Data are expressed as odds ratios (ORs) with 95% credible intervals (CrIs). The distribution of random effects is visualised in appendix 2 (p 33). Separate variable selection and model fit procedures were carried out for ESBL *E coli* and ESBL *K pneumoniae*. Individual and household variables identified through pre-screening were included alongside region and a random intercept per individual, nested within a random intercept per household. The wet season is defined as Nov–April and dry as May–Oct. ESBL=extended spectrum β-lactamase.

We then used mixed-effect logistic regression models to identify regional differences in human ESBL-producing Enterobacterales colonisation. Variable selection resulted in 24 fixed-effect predictor variables for ESBL-producing *E coli* and 14 fixed-effect predictor variables for ESBL-producing *K pneumoniae* (appendix 2 pp 19–22)*,* as well as individual and household random effects. Here, the key risk associated with human colonisation with both ESBL-producing *E coli* (figure 4A) and ESBL-producing *K pneumoniae* (figure 4B) was the wet season (aOR for *E coli* 1·66, 95% CrI 1·38–2·00, and for *K pneumoniae* 2·12, 1·63–2·76).

Bacterial species-specific risks other than the wet season were also identified. Human ESBL-producing *E coli* colonisation was associated with advanced age (aOR 1·14 per unit increase on log scale, 95% CrI 1·05–1·25), households where animals were kept inside the house (1·58, 1·00–2·43), and households where animals were observed interacting with food (1·62, 1·17–2·28; figure 4A). Accounting for these factors did not fully explain the increased urban EBSL *E coli* prevalence, and thus living in the urban environment remained a risk compared with the peri-urban site (aOR 2·01, 95% CrI 1·26–3·24).

Human ESBL-producing *K pneumoniae* colonisation was only shown to be associated with the wet season (figure 4B). There was no increased risk associated with urbanisation level identified for ESBL-producing *K pneumoniae* colonisation, and antibiotic usage was not associated with either ESBL-producing *E coli* or ESBL-producing *K pneumoniae* colonisation.

To explore the possibility that the difference in ESBL-producing Enterobacterales prevalence between urban, peri-urban, and rural households could be explained by covariates exerting a different effect in different regions, we fit models with a covariate by region interaction (appendix 2 pp 24–27); however, the only covariate that exerted a varying effect size across regions was that of seasonality.

## Discussion

In this large, One Health, longitudinal cohort study, we have identified extremely high levels of ESBL-producing Enterobacterales colonisation in humans and animals alongside extensive ESBL-producing Enterobacterales contamination of the environments in urban, peri-urban, and rural Malawi. We have described the paucity of household environmental health infrastructure and noted variations between sites. Further, we have highlighted that the key risks associated with human ESBL-producing Enterobacterales colonisation are linked to environmental sanitation, urbanisation, and the wet season.

Antibiotic use is increasing globally, both in human health and livestock production, and this has been highlighted by many authorities as an important driver of AMR.^14^, ^15^ In Malawi, antibiotic use in animals is often limited due to cost,^16^ with the exception of households that engage in small-scale intensive farming in urban settings.^17^ Consistent with findings in other settings in sub-Saharan Africa, we found moderate levels of human antibiotic use in the community, with drugs of narrow antimicrobial spectrum,^15^ and low levels of antibiotic use in household animals, but very high rates of ESBL-producing Enterobacterales colonisation. Local animal husbandry practices, including proximity to and location of animal cohabitation, household attitudes to animal and human waste management in the shared environment, and animal interactions with key external environments, are likely to promote ESBL acquisition.^18^, ^19^, ^20^ Evidence from other African settings illustrates that sharing of ESBL-producing bacteria between household members and domestic animals and livestock is commonplace.^21^, ^22^, ^23^, ^24^, ^25^, ^26^ We found animals were regularly in contact with heavily contaminated external environments (open drains and rivers) and also with household food and food preparation areas, which was associated with higher odds of ESBL-producing *E coli* colonisation in humans. Additionally, animals, especially poultry, were frequently kept inside the household at increased risk to residents and animal and human waste was commonly identified in or around the household. These animal–human–waste interactions probably drive the maintenance and transmission of ESBL-producing Enterobacterales within animals, especially in urban settings.

There was a paucity of environmental health infrastructure, a scarcity of access to hygiene materials, and high-risk behavioural proxies for faecal-oral acquisition at a household level. These behavioural proxies included frequent interactions with open drains and rivers within the local vicinity, where there are consistently high levels of ESBL-producing Enterobacterales likely derived from inadequate human and animal waste management. Our data therefore point to unrestricted shedding of human and animal waste into an unprotected environment as playing a key role in AMR transmission, whether acquisition is from household members (ie, human–human transmission), co-located animals (ie, human–animal transmission or vice versa), or transmission to and from the external environment. We propose that availability and quality of environmental health infrastructure and services, hygiene practices, and environmental hygiene govern the transmission of ESBL-producing Enterobacterales in Malawian communities.

Our study has limitations; the majority of our demographic, antibiotic use, animal husbandry data, and some environmental health data were self-reported by participants and subject to recall bias.^27^ To mitigate this potential bias, where possible, we used observed data from checklists and implemented a modified drug-bag method.^11^, ^28^ Irrespective, antibiotics can often be misidentified^28^ or ingested without knowledge,^29^ affecting the accuracy of antibiotic usage data. Not all individuals within households consented to participate, and the effect of information loss from these individuals is unknown. Lastly, the AMR data presented are phenotypic and ESBL production is inferred based on screening by ESBL-CHROMagar media. Future whole genome sequencing of the archive from this study is underway to permit transmission modelling to be undertaken, and these data will be integrated into agent-based models to determine how best to interrupt transmission of specific lineages of ESBL-producing Enterobacterales in this setting.

In conclusion, we found troublingly high prevalence of ESBL-producing Enterobacterales colonisation in humans and animals, together with extensive ESBL-producing Enterobacterales contamination of households and the broader environment (ie, rivers and drains), in southern Malawi. Our findings also highlight the key role that environmental health infrastructure and interaction with a contaminated environment has on driving human community carriage of ESBL-producing Enterobacterales, especially in urban settings. We therefore propose that without adequate efforts to reduce ESBL-producing Enterobacterales contamination of the shared environment, both at a household and community level, we are unlikely to be able to control ESBL-producing Enterobacterales transmission in Malawi and similar settings across east Africa. Lastly, regional differences in AMR prevalence exist, influenced by region-specific environmental health and animal husbandry factors. Therefore, future interventions aimed at interrupting ESBL-producing Enterobacterales transmission should be tailored in response to national AMR surveillance data.

## Data sharing

All relevant data are included in the manuscript and supplementary data, alongside de-identified participant, household, and laboratory data used in the modelling in appendix 3.

## Declaration of interests

We declare no competing interests.

## Supplementary Materials

Chichewa translation of the abstract
