# Supplementary material for: Investigating One Health risks for human colonisation with extended spectrum β-lactamase-producing Escherichia coli and Klebsiella pneumoniae in Malawian households: a longitudinal cohort study
Source: Lancet Microbe. 2023 Jul;4(7):e534–43. doi: 10.1016/S2666-5247(23)00062-9 (PMC10319635; doi:10.1016/S2666-5247(23)00062-9)
Supplement: Chichewa translation of the abstract [file mmc1.pdf]

THE LANCET
Microbe

Supplementary appendix 1

This translation in Chichewa was submitted by the authors and we reproduce it as supplied. It has not been peer reviewed. *The Lancet*'s editorial processes have only been applied to the original in English, which should serve as reference for this manuscript.

Kutanthauzila kwa mu Chichewa uku kwachokela kwa alembi a nkhaniyi ndipo tayipeleka kwa inu m'mene tinayilandilila. Mawu a mu Chichewa sanawunikilidwenso kapena kukonzedwa. Nkhani ya mu Chingelezi yokha ndi imene yadutsa mu ukonzi wa Lancet, kotelo kuti nkhani ya mu Chingeleziyi ndi imene ikuyimilila mokwanila nkhani yonse imene yalembedwa.

Supplement to: Cocker D, Chidziwisano K, Mphasa M, et al. Investigating One Health risks for human colonisation with extended spectrum β-lactamase-producing *Escherichia coli* and *Klebsiella pneumoniae* in Malawian households: a longitudinal cohort study. *Lancet Microbe* 2023; published online May 16. https://doi.org/10.1016/S2666-5247(23)00062-9.

English version

Summary

Background Low-income countries have high morbidity and mortality from drug-resistant infections, especially from enteric bacteria such as Escherichia coli. In these settings, sanitation infrastructure is of variable and often inadequate quality, creating risks of extended-spectrum β-lactamase (ESBL)-producing Enterobacterales transmission. We aimed to describe the prevalence, distribution, and risks of ESBL-producing Enterobacterales colonisation in sub-Saharan Africa using a One Health approach.

Methods Between 29th April 2019, and 3rd December 2020, we recruited 300 households in Malawi for this longitudinal cohort study: 100 each in urban, peri-urban, and rural settings. All households underwent a baseline visit and 195 were selected for longitudinal follow-up, comprising up to three additional visits over a 6 month period. Data on human health, antibiotic usage, health-seeking behaviours, structural and behavioural environmental health practices, and animal husbandry were captured alongside human, animal, and environmental samples. Microbiological processing determined the presence of ESBL-producing E. coli and Klebsiella pneumoniae, and hierarchical logistic regression was performed to evaluate the risks of human ESBL-producing Enterobacterales colonisation.

Findings A paucity of environmental health infrastructure and materials for safe sanitation was identified across all sites. A total of 11 975 samples were cultured, and ESBL-producing Enterobacterales were isolated from 1190 (41·8%) of 2845 samples of human stool, 290 (29·8%) of 973 samples of animal stool, 339 (66·2%) of 512 samples of river water, and 138 (46·0%) of 300 samples of drain water. Multivariable models illustrated that human ESBL-producing E coli colonisation was associated with the wet season (adjusted odds ratio 1·66, 95% credible interval 1·38–2·00), living in urban areas (2·01, 1·26–3·24), advanced age (1·14, 1·05–1·25), and living in households where animals were observed interacting with food (1·62, 1·17–2·28) or kept inside (1·58, 1·00–2·43). Human ESBL-producing K pneumoniae colonisation was associated with the wet season (2·12, 1·63–2·76).

Interpretation There are extremely high levels of ESBL-producing Enterobacterales colonisation in humans and animals and extensive contamination of the wider environment in southern Malawi. Urbanisation and seasonality are key risks for ESBL-producing Enterobacterales colonisation, probably reflecting environmental drivers. Without adequate efforts to improve environmental health, ESBL-producing Enterobacterales transmission is likely to persist in this setting.

Funding Medical Research Council, National Institute for Health and Care Research, and Wellcome Trust.

Chichewa version

Zotsatira za kafuku-fukuyu mwachidule

Mau oyamba Matenda ndi imfa mmaiko osauka ndi zochuluka chifukwa cha matenda omwe amakhala opima ku mankhwala, amene amadza chifukwa cha tizilombo ta bacteria tomwe timakhala m'mimba monga *Escherichia coli (E. coli)*. Mmaikowa, chisamaliro cha malo ndi zimbudzi chimakhala choperewela, zimene zimabweretsa chiopsyeza chofalitsa tizilomboti. Cholinga cha kafukufuku wathu

chidali kuona mmene vuto la kupima kwa tizilomboti ku mankhwala lilili mmaiko osauka, chiwerengero cha anthu amene ali ndi vutoli, chiopsyezo chimene chilipo mu maiko aku m'mwera kwa Africa chothandizira kufala kwa vutoli pogwiritsa ntchito upangiri wa *One Health* pothana ndi vutoli.

Ndondomeko ya kafukufuku Kuyambira pa 29 April mu 2019 mpaka pa 3 December mu 2020, makomo 300 m'Malawi adatenga nawo mbali mu kafukufukuyu: makomo 100 anali a madera akumudzi, 100 ena makomo a mkatikati mwatauni, ndi ena 100 oyimilira madera apakatikati pa mtauni ndi kumudzi. Makomo 300 onsewo adatenga nawo mbali mu kafukufuku woyambilira ndipo nyumba 195 zidasankhidwa kuti ziyenderedwe katatu kwa miyezi isanu ndi umodzi (6). Uthenga okhudza umoyo wa anthu, kagwiritsidwe ntchito ka mankhwala a antibiotic, zikhalidwe zokhuza kufuna thandizo pamene anthu adwala, zipangizo ndi zikhalidwe zokhudzana ndi ukhondo m'malo omwe anthu amakhala, komanso kasamalidwe ka ziweto zidatengedwa. Zokayesa zina zinatengedwa kuchoka ku ziweto, anthu komanso malo ozungulira makomo. Kupezeka kwa tizilombo ta *E. coli* ndi *Klebsiella pneumonia* kunaunikidwa, komanso zidafotokozeredwa kuti zilumikizitse zochitika ndi chikhalidwe za ukhondo ngati zikuthandizira kupezeka kwa tizilombo timeneti.

Zotsatira Kuchepa kwa Zipangizo zabwino zolimbikitsa ukhondo zinapezeka kuti ndi vuto lalikulu m'madera onse. Zoyesa zokwana 11, 975 zidatoleredwa ndipo tizilombo ta ESBL tokwana 1190 tidapezeka mu zoyesa za chimbudzi cha anthu 2845 (zimene zikuimilira 41 pa 100 zilizonse), tizilombo 290 zidapezedwa mu zoyesa za chimbudzi cha ziweto 973 (zimene zikuimilira 29.8 pa 100 zilizonse), tizilombo 339 zidapezedwa mu zoyesa za madzi akumtsinje 512 (zimene zikuimilira 66.2 pa 100 zilizonse), ndi tizilombo 138 zidapezedwa mu zoyesa za zithaphwi 300 (zimene zikuimilira 46 pa 100 zilizonse). Kauniuni wa zotsatira adawonetsa kuti nyengo ya dzinja (yamvula), kukhala madera a mkatikati mwatauni, ukalamba, komanso kukhala m'makomo m'mene ziweto zimatha kukhudzana ndi chakudya, kapena kugona m'nyumba limodzi ndi anthu zikuthandizira kupezeka kwa tizilomboti mu chimbudzi cha anthu. Kauniuniyo adaonetsanso kuti padali kulumikizana pakati pa nyengo ya dzinja ndi kupezeka kwa tizilombo ta *Klesbisella pneumoniae* mwa anthu.

Kutanthauza kwa zotsatira Tizilombo ta ESBL tikupezeka kwambiri mwa anthu ndi ziweto ndipo izi zikupereka chiopsyezo chachikulu ku chigawo chakum'mwera kwa Malawi. Kukula kwa matauni komanso nyengo za pachaka zikuonjera chiopsyezo cha tizilombo ta ESBL, zomwe zikupeleka chithunzithunzi cha zinthu zobweretsa chiopsyezo zomwe zikupezeka mmalo okhala anthu. Ngati ukhondo wa anthu ndi malo ozungulira susintha, vutoli likhala likupitilirabe.

Thandizo Kafukufuyu adalandira thandizo kuchokera ku bungwe la Medical Research Council, National Institute for Health and Care Research komanso Wellcome Trust.
