# Supplementary material for: Investigating One Health risks for human colonisation with extended spectrum β-lactamase-producing Escherichia coli and Klebsiella pneumoniae in Malawian households: a longitudinal cohort study
Source: Lancet Microbe. 2023 Jul;4(7):e534–43. doi: 10.1016/S2666-5247(23)00062-9 (PMC10319635; doi:10.1016/S2666-5247(23)00062-9)
Supplement: Supplementary appendix 2 [file mmc2.pdf]

THE LANCET
Microbe

Supplementary appendix 2

This appendix formed part of the original submission and has been peer reviewed. We post it as supplied by the authors.

Supplement to: Cocker D, Chidziwisano K, Mphasa M, et al. Investigating One Health risks for human colonisation with extended spectrum β-lactamase-producing *Escherichia coli* and *Klebsiella pneumoniae* in Malawian households: a longitudinal cohort study. *Lancet Microbe* 2023; published online May 16. https://doi.org/10.1016/S2666-5247(23)00062-9.

Investigating One Health risks for human colonisation with extended spectrum beta-lactamase producing *E. coli* and *K. pneumoniae* in Malawian households: a longitudinal cohort study.

	Title	Page
**Table S1**	Regional descriptions	**2**
**Table S2a**	Overview of sampling collection methods	**3**
**Table S2b**	Overview of microbiological methods	**4**
**Table S3a**	Individual-level variables selected from the CRFs for analysis, including any groupings, outputs and transformations undertaken.	**4**
**Table S3b**	Household, WASH and sampling variables selected from the CRFs for analysis, including any outputs and transformations undertaken. Variables are grouped into reported, observed and laboratory categories and stratified by factor type.	**5**
**Table S3c**	Outcome variables and covariates.	**7**
**Table S4**	STROBE Statement	**8**
**Table S5a**	Household (baseline) ABU from urban, peri-urban and rural sites.	**11**
**Table S5b**	AMU in different age groups	**12**
**Table S5c**	Regional differences in AMU stratified by age group	**12**
**Table S6**	Baseline animal husbandry characteristics, stratified by region	**13**
**Table S7**	Domestic animal and livestock ownership and husbandry	**14**
**Table S8**	Healthcare choices for household animals	**15**
**Table S9**	Baseline environmental health infrastructure, practices and environmental exposures	**16**
**Table S10**	Numbers of samples screened for ESBL *E. coli* and ESBL *K. pneumoniae*, stratified by sample type and region.	**18**
**Table S11**	Seasonal variations in ESBL prevalence of household samples.	**18**
**Table S12a**	Regional univariate analysis of WASH and individual variables against human ESBL *E. coli* colonisation	**19**
**Table S12b**	Regional univariate analysis of WASH and individual variables against human ESBL *K. pnuemoniae* colonisation	**21**
**Table S13a**	Odds ratios for covariates in ESBL *E. coli* model.	**23**
**Table S13b**	Odds ratios for covariates in ESBL *K. pnuemoniae* model.	**23**
**Table S14a**	Table of parameter testing for regional adjustment of variables included in the ESBL *E. coli* mixed effects model	**24**
**Table S14b**	Table of parameter testing for regional adjustment of variables included in the ESBL *K. pnuemoniae* mixed effects model	**25**

**Fig S1a**	Parameter estimates for the fixed-effects used in a multivariable model of ESBL *E. coli* colonisation, expressed as odds ratios with 95% CrI.	**26**
**Fig S1b**	Parameter estimates for the fixed-effects used in a multivariable model of ESBL *K. pneumoniae* colonisation, expressed as odds ratios with 95% CrI.	**27**
**Fig S2**	Bubble plot of ESBL-E prevalence in animal stool samples	**28**
**Fig S3a**	Facet Plot showing flux of human ESBL (*E. coli* or *K. pneumoniae*) colonisation amongst urban household members over time, grouped by the 65 households recruited.	**29**
**Fig S3b**	Facet Plot showing flux of human ESBL (*E. coli* or *K. pneumoniae*) colonisation amongst peri-urban household members over time, grouped by the 65 households recruited.	**30**
**Fig S3c**	Facet Plot showing flux of human ESBL colonisation (*E. coli* or *K. pneumoniae*) amongst rural household members over time, grouped by the 65 households recruited.	**31**
**Fig S4a**	PCA analysis of individual variables	**32**
**Fig S4b**	PCA analysis of household variables	**32**
**Fig S4c**	PCA analysis of environmental contamination variables	**33**
**Fig S5**	Random effects from Bayesian multivariate models of (a) ESBL *E. coli* [ESBL-E], and (b) ESBL *K. pnuemoniae* [ESBL-K], inclusive of within household (hid) and within participant (hid:pid) effects.	**33**

S1 Table. Regional descriptions

Polygon	Site Description	Access to Healthcare
Ndirande (urban site) *Size: 3 km^2*	Ndirande is a large urban settlement with high-density housing, 4 km from the geographical centre of Blantyre.<br><br><u>Animal husbandry</u><br>Small-scale intensive-farming of poultry has been increasingly seen in this region.	Healthcare is provided by local health care centres alongside access to a tertiary hospital in Blantyre (Queen Elizabeth Central Hospital), situated 2–6 km away.
Chileka (peri-urban site) *Size: 14 km^2*	Chileka is a peri-urban administrative ward on the northern outskirts of the city, with a mixture of industry, commercial farming and housing.<br><br><u>Animal husbandry</u><br>Livestock production includes a combination of large commercial farms (beef and pork) and small-scale intensive-farming.	Healthcare is provided by local health care centres, a small local private hospital (Mtengo-Umodzi) and the tertiary hospital in Blantyre (Queen Elizabeth Central Hospital), situated 10–16 km away.
Chikwawa (rural site) *Size: 71 km^2*	Chikwawa is a rural district situated in the southern Shire valley, roughly 50 km from Blantyre. There is a mixture of subsistence and large-scale sugar farms, and given its low-lying situation is historically prone to flooding<br><br><u>Animal husbandry</u><br>Sporadic large-scale cattle (beef/dairy) farming.	Healthcare is provided by local health care centres alongside access to Chikwawa District Hospital, and the tertiary hospital in Blantyre (Queen Elizabeth Central Hospital) as required.

S2a Table. Overview of sampling collection methods and numbers of samples per visit

Sample type	Number of samples per visit	Collection method
Human stool	We will ask **all** household members who consented to be recruited into the study for a sample of their stool at each visit	A 30ml stool pot will be given to participants and the study team will teach them how to use it.<br>We will then collect the stool pot the following day (i.e. within 24hrs) from the house<br>In situations where a stool pot cannot be used, a rectal swab will be taken instead
Animal stool	Up to 4 animal stool samples will be taken at each visit, where animals are found.	For poultry a cloacal swab will be used.<br>For all other animals, the study team will collect stool in a 30ml stool pot
Water (drinking/ source/ rinse)	Up to 3 samples per visit. Source water will be taken directly from the source. Stored water will be taken from inside the household and rinse water will be taken from all household members	For water samples, 500ml of water will be collected in sterile 500 ml/1L Nalgene® BPA-free, polypropylene bottles
Food	2 samples per visit. Ideally, 1 vegetable and 1 fruit sample will be taken where available	Food samples will be placed directly into sterile Whirl-Pak® bags
Clothing	1 sample per visit	Samples will be collected with 3M™ Sponge-Sticks containing 10 ml of sterile buffered peptone water
Household environment	Up to 3 samples per visit	Household environmental sampling will be informed by the WASH observations to determine high-risk areas of environmental contamination. Samples will be collected using 3M™ Sponge-Sticks (containing 10 ml of sterile buffered peptone water).
Household floor	1 sample per visit	Boot socks will be worn by study team and these will subsequently be placed into sterile Whirl-Pak® bags
Drain	1 sample per visit	Drain fluid will be collected in 30ml universal containers.
River water	1 sample per visit	500ml of river water will be collected in sterile 500 ml/1L Nalgene® BPA-free, polypropylene bottles
All samples will be transported to the laboratory in a cool box (2-8ºC) and processed the same day.		
For more details please see https://doi.org/10.5281/zenodo.5855773 (DRUM Collection SOP1_V2 Household Human Stool Sampling, DRUM Collection SOP2_V2 Household Animal Stool Sampling, and DRUM Collection SOP1_V2 Household Environmental Sampling).		

S2b Table. Overview of microbiological methods, sample storage and internal quality assurance

**Pre-enrichment**	All samples will be pre-enriched in buffered peptone water in an aerobic incubator at 37ºC for 18-24 hours.
**ESBL identification**	ESBL will be identified using ESBL CHROMagar™<br>- For each sample, a 1µl microbiological loop of incubated BPW will be streaked on a chromogenic agar plate.<br>- Chromogenic agar plates will then be incubated in an aerobic incubator at ºC for 18-24 hours and read for evidence of growth.<br>- Pink colonies will be classified as ESBL *E. coli*<br>- Blue colonies will undergo HRM PCR to speciate for *K. pneumoniae*, and PCR positive samples will be classified as ESBL *K. pneumoniae.*<br>- White colonies will undergo indole testing, and indole positive white colonies will be classified as ESBL *E. coli*
**Samples stored for future analysis**	- An aliquot of human and animal stool will be stored at -80˚C<br>- River water membranes will be stored at -80˚C<br>- An aliquot of BPW from all samples will be stored at -80˚C<br>- A single pick will be taken of each different colony colour from **all** ESBL CHROMagar™ plates where there is evidence of growth (i.e. if there are only pink colonies, then one pick will be taken. If there are both pink and blue colonies, one pick would be taken from each.<br>- All positive ESBL CHROMagar™ plates will have a plate sweep stored (BPW/glycerol) at -80˚C
**Quality assurance methods for ESBL CHROMagar™**	- ESBL CHROMagar™ plates will be made in batches each week, using the manufacturer set standards and guidance.<br>- Newly poured plates are to be left on the bench overnight at room temperature, to look for the presence of contamination.<br>- From each batch of ESBL plates made, 3 plates will be tested with a positive and negative control for ESBL-E, using the same microbiological processing methods used for collected samples. (Positive control = confirmed ESBL *K. pneumoniae*, Negative control = cephalosporin susceptible *Salmonella enterica*.)<br>- If any plates or batches do not pass quality checks, they will be discarded.<br>- Once quality checks have been completed, the plates can be stored at 2-8 degrees until used, and not kept for longer than 14 days.
For more details please see https://doi.org/10.5281/zenodo.5855773 (DRUM_SOP3_V3 ESBL culture).	

S3a Table. Individual-level variables selected from the CRFs for analysis, including any groupings, outputs and transformations undertaken.

Variable name	Description
Age*	Continuous, age at enrolment (years)
Male	Binary, {1 = male, 0 = female}
Religion (Christianity)	Binary, {1 = faith reported as Christianity, 0 = other religion practiced}
School (attendance)	Binary, {1 = attend school, 0 = do not attend school}
School (work)	Binary, {1 = work in/at school, 0 = do not work in/at school}
Healthcare (work)	Binary, {1 = work in/at hospital, 0 = do not work in/at hospital}
Hospital admission	Binary, {1 = admitted overnight to hospital in last 6 months, 0 = not admitted over last 6 months}
Hospital guardian	Binary, {1 = been a guardian at hospital in last 6 months, 0 = not been a guardian at hospital in last 6 months)
Employed	Binary, {1 = have regular job at recruitment, 0 = no job at recruitment}
Residency	Binary, {1 = resident for year or more at household, 0 = not resident for year}
Travel (Outside region)	Binary, {1 = travel outside region (any purpose in last 6 months, 0 = no travel outside area in last 6 months}
HIV status	Binary, {1 = HIV reactive, 0 = HIV non-reactive or unknown}
TB history	Binary, {1 = ever had diagnosis of TB, 0 = never had TB}
Comorbidities	Binary, {1 = 1 or more comorbidities, 0 = no comorbidities}

Medication	Binary, {1 = take any prescribed regular medication, 0 = do not take any regular medication}
Unwell 4 weeks	Binary, {1 = 1 or more unwell episodes in last 4 weeks, 0 = no illness episodes in last 4 weeks}
Unwell 3 months	Binary, {1 = 1 or more unwell episodes in last 3 months, 0 = no illness episodes in last 3 months}
ABU	Binary, {1 = 1 or more antibiotic courses taken in the last 6 months, 0 = no antibiotics taken in last 6 months}

**log-transformed*

S3b Table. Household, WASH and sampling variables selected from the CRFs for analysis, including any outputs and transformations undertaken. Variables are grouped into reported, observed and laboratory categories and stratified by factor type.

		Variable	Description
Household Factors	Reported	Number of people living in house*	Continuous, number of people cohabiting at baseline.
		Household income*	Continuous, household income (MK) at baseline.
	Lab	Share household with ESBL colonised humans	Binary, {1 = yes [share with 1 or more ESBL colonised individuals within the same household], 0 = no [Do not share with ESBL colonised household members]}
Sanitation Factors	Observed	Presence of drop hole cover	Binary, {1 = drop hole cover present, 0 = drop hole cover absent}
		Cleansing materials at toilet	Binary, {1 = cleansing material [any type] present, 0 = cleansing material [any type] absent}
		Visible human defecation	Binary, {1 = visible human stool [adult or child], 0 = no visible human stool}
	Reported	Use of pit latrine	Binary, {1 = use pit latrine, 0 = use other toilet type, or do not have toilet}
		Toilet presence (any) at household	Binary, {1 = toilet present, 0 = toilet absent}
		Open human defecation	Binary, {1 = open defecation reported [by 1 or more household members], 0 = no open defecation reported [by all household member]}
		Sharing household toilet with non-household members	Binary, {1 = shared toilet used, 0 = do not share their toilet external to the household}
		Absence of disposal mechanism for animal waste	Binary, {1 = no disposal mechanism for animal faeces, 0 = dispose of animal faeces by either sweeping them away, putting into a refuse pit or re-using as manure}
	Lab	Household environmental ESBL contamination	Binary, {1 = yes [at any point in the household during study], 0 = no [at all points during study]}
Hygiene factors	Reported	Facilities for hand washing (all areas) at household	Binary, {1 = yes [present at one or more place within the household], 0 = no [present at no places within the household]}
	Observed	Presence of soap at (any) HWF	Binary, {1 = yes [present at one or more HWFs within the household], 0 = no [present at no HWFs within the household]}
	Lab	Household rinse water ESBL contamination	Binary, {1 = yes [at any point in the household during study], 0 = no [at all points during study]}

Food Factors	Reported	Eat street food	Binary, {1 = yes [supplement diet with street food at some points], 0 = no [never buy street food]}
		Eat from shared plates	Binary, {1 = yes [use shared plates], 0 = no [do not use shared plates]}
		Buy vegetables or fruit from the market	Binary, {1 = yes [use vegetables or fruit from the market on any occasion], 0 = no [do not use market food or fruit]}
	Lab	Household food ESBL contamination	Binary, {1 = yes [at any point in the household during study], 0 = no [at all points during study]}
Water Factors	Observed	Is water stored in the house covered?	Binary, {1 = yes [water stored at the house covered], 0 = no [water not stored at the house covered]}
	Reported	Is water stored in the house?	Binary, {1 = yes [water stored at the house], 0 = no [water not stored at the house]}
		Drinking water source piped into household	Binary, {1 = yes [water source from outside the household], 0 = no [water source from pipe inside or directly outside the household]}
		Drinking water source kiosk	Binary, {1 = yes [water source from outside the household], 0 = no [water source from pipe inside or directly outside the household]}
		Drinking water source tubewell	Binary, {1 = yes [water source from outside the household], 0 = no [water source from pipe inside or directly outside the household]}
		Alternative water used for cleaning utensils	Binary, {1 = yes [different water used for cleaning utensils than for drinking], 0 = no [same water used for cleaning utensils as drinking]}
	Lab	Household source water ESBL contamination	Binary, {1 = yes [at any point in the household during study], 0 = no [at all points during study]}
		Household stored water ESBL contamination	Binary, {1 = yes [at any point in the household during study], 0 = no [at all points during study]}
Animal Factors	Observed	Animal faeces seen around the area	Binary, {1 = yes [any animal faeces seen around the household at any point], 0 = no [no animal faeces ever seen around the household]}
		Evidence of animal contact with food	Binary, {1 = yes [animal seen in contact with food], 0 = no [no animal seen in contact with food]}
	Reported	Does the household own any animals?	Binary, {1 = yes [household owns 1 or more animals], 0 = no [no animals owned by household]}
		Own cattle or ruminant	Binary, {1 = yes [household owns 1 or more animals], 0 = no [no animals owned by household]}
		Own poultry	Binary, {1 = yes [household owns 1 or more animals], 0 = no [no animals owned by household]}
		Own pet / companion animal	Binary, {1 = yes [household owns 1 or more animals], 0 = no [no animals owned by household]}
		Own pigs	Binary, {1 = yes [household owns 1 or more animals], 0 = no [no animals owned by household]}
		Animals (any species) kept inside the house?	Binary, {1 = yes [if animals owned - they kept inside the house], 0 = no [if animals owned - they are not kept inside the house]}
	Lab	Household animal ESBL contamination	Binary, {1 = yes [at any point in the household animals during study], 0 = no [at all points during study]}
Broader Environment	Observed	Accumulation of water / wastewater (household environment)	Binary, {1 = yes [water seen external to the household], 0 = no [water not seen external to the household]}

	**Reported**	Household member interaction with river water	Binary, {1 = yes [any adult or child at the household reportedly interact with river water], 0 = no [no adult or child at the household even interact with river water]}
		Household member interaction with drains	Binary, {1 = yes [any adult or child at the household reportedly interact with drains], 0 = no [no adult or child at the household even interact with drains]}
	**Lab**	Drain ESBL contamination	Binary, {1 = yes [at any point during study], 0 = no [at all points during study]}
		River ESBL contamination	Binary, {1 = yes [at any point during study], 0 = no [at all points during study]}

**log-transformed*

S3c Table. Outcome variables and covariates.

Dependant variable	Description
ESBL (positive)	Binary, {1 = ESBL positive at single episode [with either KPN or EC], 0 = ESBL negative at single episode [with either KPN or EC]}
ESBL-E (positive)	Binary, {1 = ESBL *E. coli* positive at single episode, 0 = ESBL *E. coli* negative at single episode}
ESBL-K (positive)	Binary, {1 = ESBL *K. pneumoniae* positive at single episode, 0 = ESBL *K. pneumoniae* negative at single episode}
**Covariates**	
Region	Categorical, {Urban / Peri-urban / rural}
Season	Binary, {1 = wet (October-April), 0 = dry (May-September)}

S4 Table: STROBE Statement

	Item No	Recommendation	Page No
**Title and abstract**	1	(*a*) Indicate the study's design with a commonly used term in the title or the abstract	1 (title)
		(*b*) Provide in the abstract an informative and balanced summary of what was done and what was found	3
**Introduction**			
Background/rationale	2	Explain the scientific background and rationale for the investigation being reported	4 (research in context) and 7 (introduction)
Objectives	3	State specific objectives, including any prespecified hypotheses	8
**Methods**			
Study design	4	Present key elements of study design early in the paper	8-10, including referenced links to online protocol and affiliated CRFs/SOPs
Setting	5	Describe the setting, locations, and relevant dates, including periods of recruitment, exposure, follow-up, and data collection	8 and Appendix S1. Also, setting and location described in more detail in protocol paper which is referenced in the text.
Participants	6	(*a*) *Cohort study*—Give the eligibility criteria, and the sources and methods of selection of participants. Describe methods of follow-up<br>*Case-control study*—Give the eligibility criteria, and the sources and methods of case ascertainment and control selection. Give the rationale for the choice of cases and controls<br>*Cross-sectional study*—Give the eligibility criteria, and the sources and methods of selection of participants	8-9
		(*b*) *Cohort study*—For matched studies, give matching criteria and number of exposed and unexposed<br>*Case-control study*—For matched studies, give matching criteria and the number of controls per case	NA
Variables	7	Clearly define all outcomes, exposures, predictors, potential confounders, and effect modifiers. Give diagnostic criteria, if applicable	9-10 and Appendix S2a-c Tables.
Data sources/ measurement	8*	For each variable of interest, give sources of data and details of methods of assessment (measurement). Describe comparability of assessment methods if there is more than one group	S2a-c Tables. Statistical methods explained in pages 9-10. Data included in S3a-e
Bias	9	Describe any efforts to address potential sources of bias	8-10 and 19.
Study size	10	Explain how the study size was arrived at	8, and detailed in protocol paper referenced in the manuscript.

Quantitative variables	11	Explain how quantitative variables were handled in the analyses. If applicable, describe which groupings were chosen and why	S2a-c Tables. Statistical methods explained in pages 8-10.
Statistical methods	12	(*a*) Describe all statistical methods, including those used to control for confounding	8-10
		(*b*) Describe any methods used to examine subgroups and interactions	8-10 and S1a-b figs
		(*c*) Explain how missing data were addressed	8-10
		(*d*) *Cohort study*—If applicable, explain how loss to follow-up was addressed<br>*Case-control study*—If applicable, explain how matching of cases and controls was addressed<br>*Cross-sectional study*—If applicable, describe analytical methods taking account of sampling strategy	8 and protocol paper
		(*e*) Describe any sensitivity analyses	NA

Continued on next page

**Results**			
Participants	13*	(a) Report numbers of individuals at each stage of study—eg numbers potentially eligible, examined for eligibility, confirmed eligible, included in the study, completing follow-up, and analysed	11
		(b) Give reasons for non-participation at each stage	11 and fig 1
		(c) Consider use of a flow diagram	11 and fig 1
Descriptive data	14*	(a) Give characteristics of study participants (eg demographic, clinical, social) and information on exposures and potential confounders	11-12 and appendix.
		(b) Indicate number of participants with missing data for each variable of interest	NA
		(c) *Cohort study*—Summarise follow-up time (eg, average and total amount)	Fig 1
Outcome data	15*	*Cohort study*—Report numbers of outcome events or summary measures over time	15-16 and appendix
		Case-control study—Report numbers in each exposure category, or summary measures of exposure	NA
		Cross-sectional study—Report numbers of outcome events or summary measures	NA
Main results	16	(*a*) Give unadjusted estimates and, if applicable, confounder-adjusted estimates and their precision (eg, 95% confidence interval). Make clear which confounders were adjusted for and why they were included	11-17.
		(*b*) Report category boundaries when continuous variables were categorized	11-12, 15-16,
		(*c*) If relevant, consider translating estimates of relative risk into absolute risk for a meaningful time period	NA
Other analyses	17	Report other analyses done—eg analyses of subgroups and interactions, and sensitivity analyses	Supplementary data
**Discussion**			
Key results	18	Summarise key results with reference to study objectives	17
Limitations	19	Discuss limitations of the study, taking into account sources of potential bias or imprecision. Discuss both direction and magnitude of any potential bias	19
Interpretation	20	Give a cautious overall interpretation of results considering objectives, limitations, multiplicity of analyses, results from similar studies, and other relevant evidence	18-19
Generalisability	21	Discuss the generalisability (external validity) of the study results	19
**Other information**			
Funding	22	Give the source of funding and the role of the funders for the present study and, if applicable, for the original study on which the present article is based	Listed during submission

S5a Table. Household (baseline) ABU from urban, peri-urban and rural sites.

Variable	Site	Total antibiotics	Antibiotic choice^										
			Amoxicillin	Benzylpenicillin	Cefuroxime	Cefixime	Ciprofloxacin	Co-trimoxazole	Doxycycline	Erythromycin	Gentamicin	Metronidazole	Other or Unknown
**Antibiotic usage in household participants**	Urban	n=63	n=24 (38.1%)	n=2 (3.2%)	n=0 (0.0%)	n=0 (0.0%)	n=2 (3.2%)	n=20 (31.7%)	n=0 (0.0%)	n=2 (3.2%)	n=3 (4.8%)	n=7 (11.1%)	n=3 (4.8%)
	Peri-urban	n=40	n=9 (22.5%)	n=0 (0.0%)	n=0 (0.0%)	n=0 (0.0%)	n=3 (7.5%)	n=17 (42.5%)	n=0 (0.0%)	n=1 (2.5%)	n=1 (2.5%)	n=8 (20.0%)	n=1 (2.5%)
	Rural	n=78	n=31 (39.7%)	n=1 (1.3%)	n=1 (1.3%)	n=1 (1.3%)	n=2 (2.6%)	n=28 (35.9%)	n=2 (2.6%)	n=1 (1.3%)	n=2 (2.6%)	n=8 (10.3%)	n=1 (1.3%)
**Antibiotic used in last 4 weeks**	Urban	n=21	n=5 (23.8%)	n=0 (0.0%)	n=0 (0.0%)	n=0 (0.0%)	n=1 (4.8%)	n=10 (47.6%)	n=0 (0.0%)	n=0 (0.0%)	n=1 (4.8%)	n=2 (9.5%)	n=2 (9.5%)
	Peri-urban	n=24	n=4 (16.7%)	n=0 (0.0%)	n=0 (0.0%)	n=0 (0.0%)	n=1 (4.2%)	n=12 (50.0%)	n=0 (0.0%)	n=0 (0.0%)	n=1 (4.2%)	n=5 (20.8%)	n=1 (4.2%)
	Rural	n=29	n=12 (41.4%)	n=0 (0.0%)	n=0 (0.0%)	n=0 (0.0%)	n=1 (3.4%)	n=12 (41.4%)	n=0 (0.0%)	n=0 (0.0%)	n=0 (0.0%)	n=3 (10.3%)	n=1 (3.4%)
**Antibiotic used in last 4 weeks to 3 months**	Urban	n=24	n=8 (33.3%)	n=1 (4.2%)	n=0 (0.0%)	n=0 (0.0%)	n=1 (4.2%)	n=6 (25.0%)	n=0 (0.0%)	n=2 (8.3%)	n=1 (4.2%)	n=4 (16.7%)	n=1 (4.2%)
	Peri-urban	n=8	n=3 (32.5%)	n=0 (0.0%)	n=0 (0.0%)	n=0 (0.0%)	n=1 (12.5 %)	n=2 (25.0%)	n=0 (0.0%)	n=1 (12.5 %)	n=0 (0.0%)	n=1 (12.5%)	n=0 (0.0%)
	Rural	n=33	n=12 (36.4%)	n=1 (3.0%)	n=1 (3.0%)	n=0 (0.0%)	n=1 (3.0%)	n=11 (33.3%)	n=2 (6.0%)	n=0 (0.0%)	n=2 (6.0%)	n=3 (9.1%)	n=0 (0.0%)
**Antibiotic used in last 6 months* (healthcare)**	Urban	n=15	n=9 (60.0%)	n=1 (6.7%)	n=0 (0.0%)	n=0 (0.0%)	n=0 (0.0%)	n=3 (20.0%)	n=0 (0.0%)	n=0 (0.0%)	n=1 (6.7%)	n=1 (6.7%)	n=0 (0.0%)
	Peri-urban	n=3	n=0 (0.0%)	n=0 (0.0%)	n=0 (0.0%)	n=0 (0.0%)	n=1 (33.3 %)	n=1 (33.3%)	n=0 (0.0%)	n=0 (0.0%)	n=0 (0.0%)	n=1 (33.3%)	n=0 (0.0%)
	Rural	n=15	n=7 (46.7%)	n=0 (0.0%)	n=0 (0.0%)	n=1 (6.7%)	n=0 (0.0%)	n=4 (26.7%)	n=0 (0.0%)	n=1 (6.7%)	n=0 (0.0%)	n=2 (13.3%)	n=0 (0.0%)
**Antibiotic used at baseline**	Urban	n=3	n=2 (66.7%)	n=0 (0.0%)	n=0 (0.0%)	n=0 (0.0%)	n=0 (0.0%)	n=1 (33.3%)	n=0 (0.0%)	n=0 (0.0%)	n=0 (0.0%)	n=0 (0.0%)	n=0 (0.0%)
	Peri-urban	n=5	n=2 (40.0%)	n=0 (0.0%)	n=0 (0.0%)	n=0 (0.0%)	n=0 (0.0%)	n=2 (40.0%)	n=0 (0.0%)	n=0 (0.0%)	n=0 (0.0%)	n=1 (20.0%)	n=0 (0.0%)
	Rural	n=1	n=0 (0.0%)	n=0 (0.0%)	n=0 (0.0%)	n=0 (0.0%)	n=0 (0.0%)	n=1 (100%)	n=0 (0.0%)	n=0 (0.0%)	n=0 (0.0%)	n=0 (0.0%)	n=0 (0.0%)

*^Grey = Total usage, where antibiotics were selected by ≥1 households in region. Blue = cumulative total of antibiotics used. Yellow = antibiotic selected by ≥1 households in region. White = antibiotic not selected.*Provided to participant at healthcare services during an acute presentation.*

S5b Table. AMU in different age groups

Reported ABU		n (%)			
**Antibiotic use by age group[$]**		**Child**	**Adolescent**	**Adult**	*p*
Antibiotic usage (total all regions)	NA	49 (32.5%)	39 (11.9%)	59 (12.2%)	***>.001***

[$]Total Adult (>17) = 485, Adolescents (5-17) = 329, and Children <5 = 151

S5c Table. Regional differences in AMU stratified by age group.

Reported ABU	n (%)			
**Antibiotic use by age group[$]**	**Urban**	**Peri-urban**	**Rural**	*p*
Child (<5)	n=17 (32.7%)	n=11 (21.6%)	n=21 (43.8%)	*.06*
Adolescent (5-17)	n=15 (12.9%)	n=11 (9.0%)	n=13 (14.3%)	*.45*
Adult (>17)	n=19 (13.1%)	n=13 (6.2%)	n=27 (20.6%)	***>.001***

[$]Total Adult (>17) = 485, Adolescents (5-17) = 329, and Children <5 = 151

S6 Table. Baseline animal husbandry characteristics, stratified by region

	n (%)				
	**Total** *(n=300)*	**Urban** *(n=100)*	**Peri-urban** *(n=100)*	**Rural** *(n=100)*	***p***
Animal husbandry					
**Households that own animals**	176 (58.7%)	36 (36%)	59 (59%)	81 (81%)	***<.001***
**Species of animals owned^**					
Chickens	116 (38.7%)	18 (18.0%)	39 (39.0%)	59 (59.0%)	***<.001***
Goats	49 (16.3%)	0 (0.0%)	12 (12.0%)	37 (37.0%)	***<.001***
Dogs	43 (14.3%)	14 (14.0%)	19 (19.0%)	10 (10.0%)	*.212*
Cats	24 (8.0%)	9 (9.0%)	6 (6.0%)	7 (7.0%)	*.790*
Cattle	23 (7.7%)	0 (0.0%)	0 (0.0%)	23 (23.0%)	*NA*
Pigs	17 (5.7%)	0 (0.0%)	5 (5.0%)	12 (12.0%)	***<.001***
Ducks	14 (4.7%)	2 (2.0%)	2 (2.0%)	10 (10.0%)	***.017***
Doves	13 (4.3%)	1 (1.0%)	5 (5.0%)	7 (7.0%)	*.092*
Other	6 (2.8%)	2 (2.4%)	3 (3.5%)	1 (2.4%)	*1.00*
Guinea fowl	3 (1.0%)	0 (0.0%)	0 (0.0%)	3 (3.0%)	*.109*
Turkeys	2 (0.7%)	0 (0.0%)	2 (2.0%)	0 (0.0%)	*.331*
**Households that rear animals to sell?**	77 (25.7%)	3 (3.0%)	25 (25.0%)	49 (49.0%)	***<.001***
**Chickens kept inside the house, yes***	70 (60.3%)	15 (83.3%)	25 (64.1%)	30 (50.8%)	***.041***
**Goats kept inside the house, yes***	8 (16.3%)	NA	5 (41.7%)	3 (8.1%)	***.015***
Animal disease and antibiotic usage					
**Disease noted in last 12 months, yes**					
Poultry	51 (44.3%)	8 (44.4%)	17 (44.7%)	26 (44.0%)	
Cattle	8 (34.8%)	NA	NA	8 (34.8%)	
Pigs	4 (23.5%)	NA	0 (0.0%)	4 (33.3%)	
Goats	11(22.4%)	NA	1 (8.3%)	10 (27.0%)	
**Access to professional animal health services**	47 (26.9%)	7(19.4%)	11 (19.0%)	29 (35.8%)	*0.054*
**Antibiotics given to animals in the last 2 months**	7 (4.0%)	1 (2.8%)	2 (3.4%)	4 (4.9%)	*1.00*

**= denominator includes only those households that either owned chickens or goats.*

S7 Table. Domestic animal and livestock ownership and husbandry

**Household ownership**	**n**			
	**Total**	**Urban**	**Peri-urban**	**Rural**
**Total number of animals owned**	2169	213	704	1252
**Species of animals owned^**				
Number of chickens	919	152	315	452
Number of doves	442	10	250	182
Number of ducks	67	14	8	45
Number of guinea fowl	34	0	0	34
Number of turkeys	8	0	8	0
Number of dogs	100	27	44	29
Number of cats	31	10	11	10
Number of cattle	23	0	0	74
Number of pigs	17	0	20	55
Number of goats	419	0	48	371
**Husbandry characteristics**	**n (%)**			
**Where are animals kept?**				
**Chickens**				
In the house	70 (60.3%)	15 (83.3%)	25 (64.1%)	30 (50.8%)
Shelter /Boma within household compound	33 (28.4%)	2 (11.1%)	12 (30.8%)	19 (32.2%)
Shelter /Boma outside the household compound	9 (7.8%)	1 (5.6%)	1 (2.6%)	7 (11.9%)
Other	4 (3.4%)	0 (0.0%)	1 (2.6%)	3 (5.1%)
**Dogs**				
Free roaming	34 (79.1%)	9 (64.3%)	15 (78.9%)	10 (100.0%)
Shelter /Boma outside the household compound	1 (2.3%)	1 (7.1%)	0 (0.0%)	0 (0.0%)
Shelter /Boma within household compound	8 (18.6%)	4 (28.6%)	4 (21.1%)	0 (0.0%)
**Cattle**				
Shelter /Boma within household compound	11 (47.8%)	NA	NA	11 (47.8%)
Shelter /Boma outside the household compound	11 (47.8%)	NA	NA	11 (47.8%)
Other	1 (4.4%)	NA	NA	1 (4.2%)
**Goats**				
In the house	8 (16.3%)	NA	5 (41.7%)	3 (8.1%)
Shelter /Boma within household compound	28 (57.1%)	NA	6 (50.0%)	22 (59.5%)
Shelter /Boma outside the household compound	12 (24.5%)	NA	1 (8.3%)	11 (29.7%)
Free roaming	1 (2.0%)	NA	0 (0.0%)	1 (2.7%)
**Pigs**				
Shelter /Boma within household compound	10 (58.8%)	NA	5 (100.0%)	5 (41.7%)
Shelter /Boma outside the household compound	6 (35.3%)	NA	0 (0.0%)	6 (50.0%)
Free roaming	1 (5.9%)	NA	0 (0.0%)	1 (8.3%)
**Livestock production system**				

**Beef cattle**				
Zero Grazing	2 (%)	NA	NA	2 (10.0%)
Communal Grazing	15 (%)	NA	NA	15 (75.0%)
Pastoral	3 (%)	NA	NA	3 (15.0%)
**Dairy cattle**				
Pastoral	4 (%)	NA	NA	4 (100.0%)
**Small ruminants**				
Zero Grazing	8 (%)	NA	6 (50.0%)	2 (5.4%)
Communal Grazing	26 (%)	NA	6 (50.0%)	20 (54.1%)
Pastoral	15 (%)	NA	0 (0.0%)	15 (40.5%)

S8 Table. Healthcare choices for household animals

		Response to sickness in household animals							
**Animal**	**Site**	Consult a governmental veterinarian	Consult a private veterinarian	Use medication from a veterinarian drug store	Use left-over or vet applied drugs	Get medications from friends or family	Use traditional medication	Kill animal (+/- eaten)	Nothing
**Cattle**	Urban	NA	NA	NA	NA	NA	NA	NA	NA
	Peri-urban	NA	NA	NA	NA	NA	NA	NA	NA
	Rural	n=6	n=1	n=1	n=1	n=1	n=0	n=0	n=9
**Goats**	Urban	NA	NA	NA	NA	NA	NA	NA	NA
	Peri-urban	n=2	n=1	n=0	n=1	n=0	n=4	n=0	n=3
	Rural	n=9	n=2	n=4	n=2	n=0	n=1	n=0	n=9
**Pigs**	Urban	NA	NA	NA	NA	NA	NA	NA	NA
	Peri-urban	n=1	n=1	n=1	n=0	n=0	n=0	n=0	n=1
	Rural	n=2	n=0	n=1	n=0	n=0	n=1	n=0	n=6
**Poultry**	Urban	n=0	n=1	n=3	n=0	n=0	n=3	n=1	n=6
	Peri-urban	n=7	n=1	n=4	n=2	n=0	n=8	n=1	n=6
	Rural	n=7	n=2	n=7	n=2	n=0	n=5	n=5	n=17
**All animals**	**All regions**	n=34 (21.4%)	n=9 (5.6%)	n=21 (13.2%)	n=8 (5.0%)	n=1 (0.6%)	n=22 (13.8%)	n=7 (4.4%)	n=57 (35.8%)

^ *Yellow = selected by ≥1 households in region. White = not selected. Grey = NA.*

S9 Table. Baseline environmental health infrastructure, practices and environmental exposures

	n (%) unless otherwise indicated				
	**Total** *(n=300)*	**Urban** *(n=100)*	**Peri-urban** *(n=100)*	**Rural** *(n=100)*	***p***
Water management					
**Drinking water source**					
Tube well/ Borehole	153 (48.7%)	7 (7.0%)	60 (60.0%)	86 (86.0%)	***<.001***
Public tap/ standpipe	79 (25.2%)	64 (64.0%)	12 (12.0%)	3 (3.0%)	***<.001***
Piped outside dwelling	53 (16.9%)	22 (22.0%)	20 (20.0%)	11 (11.0%)	***.036***
Piped into dwelling	24 (7.6%)	10 (10.0%)	12 (12.0%)	2 (2.0%)	***.015***
Unprotected well /spring	2 (0.6%)	1 (1.0%)	1 (1.0%)	0 (0.0%)	*1.00*
Bottled	1 (0.3%)	0 (0.0%)	1 (1.0%)	0 (0.0%)	*1.00*
Tube well with powered pump	1 (0.3%)	0 (0.0%)	1 (1.0%)	0 (0.0%)	*1.00*
Surface water (i.e. lake / river)	1 (0.3%)	1 (1.0%)	0 (0.0%)	0 (0.0%)	*1.00*
**Drinking water treatment**	25 (8.3%)	4 (4.0%)	4 (4.0%)	17 (17.0%)	***<.001***
**Is household drinking water visibly covered?***	305 (68.1%)	120 (62.2%)	85 (62.0%)	100 (84.7%)	***<.001***
**Alternative water source used for cleaning and drinking**	52 (17.3%)	29 (29.0%)	12 (12.0%)	11 (11.0%)	***.001***
**Visible separation seen in water used for drinking and other household activities***	225 (75.5%)	74 (74.0%)	70 (70.7%)	81 (81.8%)	*.072*
Toileting, sanitation and waste management					
**Toilet present at household**	267 (89.0%)	95 (95.0%)	97 (97.0%)	75 (75.0%)	***<.001***
**Toilet type (where present)**					
Pit latrine	237 (88.8%)	90 (94.7%)	73 (75.3%)	74 (98.7%)	***<.001***
Flush toilet to septic tank	15 (5.6%)	4 (4.2%)	9 (9.3%)	0 (0.0%)	***.013***
Flush toilet to mains	7 (2.6%)	1 (1.1%)	6 (6.2%)	0 (0.0%)	***.030***
**Households who share their toilet with non-household members**	112 (41.9%)	59 (62.1%)	30 (30.9%)	23 (30.7%)	***<.001***
**Number of households toilet shared with. [median (IQR)]**	3 (2-4)	3 (2-5)	2 (2-3)	2 (1-2)	
**Households where ≥1 member practices open defecation?**	86 (28.7%)	25 (25.0%)	28 (28.0%)	33 (33.0%)	*.485*
**Human feaces present in / around the household compound? ***	66 (8.1%)	18 (6.8%)	6 (2.3%)	42 (14.7%)	***<.001***
**Drophole cover present at toilet**	92 (34.5%)	21 (22.1%)	35 (35.4%)	36 (48.0%)	***.002***
**Drophole cover in place (where available) ***	184 (73.0%)	44 (91.7%)	69(69.0%)	71 (68.3%)	***.003***
Anal cleansing materials present at toilet *	133 (18.9%)	26 (10.7%)	88 (33.4%)	19 (9.3%)	***<.001***
**Households that have an adequate system to manage animal waste?^**	13 (4.3%)	1 (1.0%)	8 (8.0%)	4 (4.0%)	*.055*
**Households that have an adequate system to manage household waste^**	24 (8.0%)	20 (20.0%)	3 (3.0%)	1 (1.0%)	***<.001***
Hand-hygiene					
**Facilities for hand washing available at households (any)***	123 (41.0%)	37 (37.0%)	63 (63.0%)	23 (23.0%)	***<.001***
**Soap (liquid/bar/powder) present at HWFs***	166 (49.0%)	28 (43.1%)	130 (58.8%)	8 (15.1%)	***<.001***
**Water present at HWFs***	349 (85.5%)	65 (71.4%)	231 (94.3%)	53 (73.6%)	***<.001***

**When do household members normally wash their hands?**					
Before eating	269 (89.7%)	90 (90.0%)	88 (88.0%)	91 (91.0%)	*.840*
After toilet	269 (89.7%)	89 (89.0%)	89 (89.0%)	91 (91.0%)	*.916*
When they look dirty	139 (46.3%)	39 (39.0%)	64 (64.0%)	36 (36.0%)	***<.001***
After eating	137 (45.7%)	74 (74.0%)	39 (39.0%)	24 (24.0%)	***<.001***
Before preparing food	110 (36.7%)	52 (52.0%)	24 (24.0%)	34 (33.0%)	***<.001***
After cleaning child nappy	67 (22.3%)	28 (28.0%)	18 (18.0%)	21 (21.0%)	*.219*
After working outside	62 (20.7%)	24 (24.0%)	12 (12.0%)	26 (26.0%)	***.027***
Before feeding child	52 (17.3%)	27 (27.0%)	12 (12.0%)	13 (13.0%)	***<.001***
Food access and hygiene					
**Consumption of market produce (vegetable)**	260 (86.7%)	98 (98.0%)	92 (92.0%)	70 (70.0%)	***<.001***
**Use of shared plates**	129 (43.0%)	28 (28.0%)	36 (36.0%)	65 (65.0%)	***<.001***
**Consumption of street food**	267 (89.0%)	93 (93.0%)	89 (89.0%)	85 (85.0%)	*.213*
**Cooked food seen to be covered***	286 (92.3%)	124 (95.4%)	77 (96.3%)	85 (85.0%)	***.007***
**Animals seen in the cooking area***	196 (24.1%)	30 (10.9%)	70 (26.4%)	96 (33.6%)	***<.001***
**Animals seen in contact with food***	123 (62.8%)	24 (80.0%)	32 (45.7%)	67 (69.8%)	***<.001***
**Utensils (covered)** *	129 (15.9%)	68 (25.9%)	53 (20.0%)	8 (2.8%)	***<.001***
**Fresh fruit and vegetables (covered)** *	131 (38.1%)	86 (50.3%)	36 (35.0%)	9 (12.9%)	***<.001***
**Meat (covered)** *	145 (84.3%)	79 (73.1%)	52 (100.0%)	8 (66.7%)	***<.001***
Environmental interactions					
**Standing water seen near the household***	65 (8.0%)	36 (13.7%)	26 (9.8%)	3 (1.0%)	***<.001***
**Children observed interacting with standing water***	16 (24.6%)	11 (30.6%)	4(15.4%)	1 (33.3%)	*.350*
**Animals observed interacting with standing water***	33 (50.8%)	18 (50.0%)	14 (53.8%)	1 (33.3%)	*.847*
**Open drains seen near the household***	137 (16.8%)	41 (15.6%)	89 (33.6%)	7 (2.4%)	***<.001***
**Children observed interacting with drains***	28 (20.4%)	11 (26.8%)	17 (19.1%)	0 (0.0%)	*.304*
**Animals observed interacting with drains***	60 (43.8%)	20 (48.8%)	35 (39.3%)	5 (71.4%)	*.205*
**Households reporting their children interacting with river water?**	66 (22.0%)	18 (18.0%)	34 (34.0%)	14 (14.0%)	***.001***
**Households reporting their adults interacting with river water?**	99 (33.0%)	26 (26.0%)	56 (56.0%)	17 (17.0%)	***<.001***

* *observed (not self-reported) results from household visits over the total study, derived as follows: [n= visits where observational data on reported activity collected (% of n visits where specific activity was observed to occur)]. ^Adequate defined as: (human waste) disposal into communal collection container, and (animal waste) removal from the premises, and subsequent contained disposal away from human contact.*

S10 Table. Numbers of samples screened for ESBL *E. coli* and ESBL *K. pneumoniae*, stratified by sample type and region.

Broad sample type		Sample number n (%)			
		**Total**	**Urban**	**Peri-urban**	**Rural**
Human stool		2845 (23.8%)	821 (22.3%)	982 (24.4%)	1042 (24.3%)
Animal stool		973 (8.1%)	118 (3.2%)	229 (5.7%)	626 (14.6%)
Environment		8157 (68.1%)	2736 (74.5%)	2807 (69.9%)	2614 (60.1%)
	Food	1168 (9.8%)	333 (9.1%)	440 (11.0%)	395 (9.2%)
	Drinking water	1254 (10.5%)	532 (14.5%)	449 (11.2%)	273 (6.4%)
	Source water	527 (4.4%)	79 (2.1%)	216 (5.4%)	232 (5.4%)
	Household surfaces	2458 (20.5%)	766 (20.8%)	744 (18.5%)	948 (22.1%)
	Household floor	745 (6.2%)	247 (6.7%)	244 (6.1%)	254 (5.9%)
	Clothing	742 (6.2%)	245 (6.7%)	242 (6.0%)	255 (5.9%)
	Hand-contact samples	451 (3.8%)	129 (3.5%)	69 (1.7%)	253 (5.9%)
	Household drains	300 (2.5%)	151 (4.1%)	149 (3.7%)	n=0 (0.0%)
	River water	512 (4.3%)	254 (6.9%)	254 (6.3%)	4 (0.1%)
**TOTAL**		**11975**	**3675**	**4018**	**4282**

S11 Table. Seasonal variations in ESBL prevalence of household samples.

	ESBL prevalence by season (%, SD)		
**Sample Type**	**Wet season** ***(Nov-Apr)***	**Dry season** ***(May-Oct)***	***p***
Human stool	47.2% (49.9)	36.6% (48.2)	***<.001***
Animal stool	33.3% (47.2)	25.5% (43.6)	***.010***
Food	14.4% (35.1)	12.3% (32.9)	*.338*
Drinking water	26.2% (44.0)	15.2% (35.9)	***<.001***
Source water	6.5% (24.7)	8.8% (28.3)	*.413*
Household surfaces	8.8% (28.3)	4.5% (20.8)	***<.001***
Household floor	11.5% (31.9)	6.6% (24.9)	***.031***
Clothing	9.1% (28.9)	5.6% (23.0)	*.087*
Hand-contact samples	25.8% (43.9)	17.9% (38.4)	*.057*
Household drains	44.7% (49.9)	48.2% (50.2)	*.648*
River water	69.1% (46.3)	62.9% (48.4)	*.164*

^p values generated by X^2 test

S12a Table. Regional univariate analysis of WASH and individual variables against human ESBL *E. coli* colonisation

Characteristic	Region	n	OR	95% CI	p value	Model inclusion
**Season (wet)**	Urban	813	1.11	0.84,1.47	0.5	**Yes**
	Peri	971	**1.92**	**1.47,2.53**	**<0.001**	
	Rural	938	1.29	0.98,1.69	0.067	
**Male sex**	Urban	813	0.82	0.61,1.09	0.2	No
	Peri	971	0.93	0.71,1.22	0.6	
	Rural	938	0.97	0.66,1.14	0.3	
**Age (log)**	Urban	813	**1.14**	**1.01,1.29**	**0.030**	**Yes**
	Peri	971	1.07	0.94,1.21	0.3	
	Rural	938	1.07	0.95,1.21	0.3	
**ABU** *(Last 6 months)*	Urban	813	1.01	0.71,1.45	>0.9	No
	Peri	971	0.89	0.57,1.37	0.6	
	Rural	938	1.15	0.83,1.57	0.4	
**HIV reactive**	Urban	813	0.85	0.48,1.49	0.6	No
	Peri	971	0.86	0.49,1.47	0.6	
	Rural	938	1.23	0.77,1.94	0.4	
**Household density (log)**	Urban	813	1.17	0.82,1.67	0.4	**Yes**
	Peri	971	0.99	0.71,1.39	>0.9	
	Rural	938	**0.66**	**0.45,0.97**	**0.034**	
**Income** *(>40,000MK/month)*	Urban	813	0.91	0.69,1.21	0.5	No
	Peri	971	1.04	0.8,1.37	0.8	
	Rural	938	0.84	0.64,1.11	0.2	
**Shared Toilet**	Urban	813	1.02	0.77,1.35	0.9	**Yes**
	Peri	971	**1.37**	**1.04, 1.81**	**0.026**	
	Rural	938	0.86	0.63,1.18	0.4	
**Drophole Present**	Urban	813	0.80	0.55, 1.15	0.2	**Yes**
	Peri	971	**0.59**	**0.44,0.79**	**<0.001**	
	Rural	938	1.08	0.81,1.44	0.6	
**Cleaning Materials available**	Urban	813	1.17	0.84,1.62	0.3	**Yes**
	Peri	971	**0.65**	**0.49,0.84**	**0.001**	
	Rural	938	0.80	0.51,1.23	0.3	
**Human Feaces visible**	Urban	813	0.95	0.72,1.25	0.7	**Yes**
	Peri	971	**1.47**	**1.08.2.01**	**0.015**	
	Rural	938	0.82	0.63,1.08	0.2	
**Human defecation practiced**	Urban	813	1.47	0.71,3.04	0.3	**Yes**
	Peri	971	1.02	0.68,1.52	>0.9	
	Rural	938	**0.59**	**0.37,0.91**	**0.021**	
**HWF present**	Urban	813	1.15	0.87,1.52	0.3	No
	Peri	971	0.74	0.53,1.06	0.1	
	Rural	938	1.07	0.82,1.40	0.6	
**Soap present**	Urban	813	**1.89**	**1.17,3.08**	**0.010**	**Yes**
	Peri	971	**0.74**	**0.56,0.98**	**0.034**	
	Rural	938	**0.47**	**0.21,0.94**	**0.044**	
**Stored water covered**	Urban	813	**2.16**	**1.38,3.47**	**<0.001**	**Yes**
	Peri	971	1.30	0.97,1.75	0.082	
	Rural	938	**0.62**	**0.46,0.81**	**<0.001**	
**Stored water covered and tap**	Urban	813	0.83	0.62,1.10	0.2	No
	Peri	971	0.76	0.46,1.21	0.3	
	Rural	938	0.76	0.57,1.01	0.056	
**Utensil water**	Urban	813	0.95	0.71,1.27	0.7	No
	Peri	971	1.43	0.93,2.18	0.10	
	Rural	938	1.22	0.84,1.74	0.3	

**Piped water (i.e. kiosk)**	Urban	813	0.97	0.73,1.29	0.9	**Yes**
	Peri	971	**0.64**	**0.47,0.88**	**0.006**	
	Rural	938	**0.68**	**0.47,0.97**	**0.036**	
**Tap water (i.e. household tap)**	Urban	813	0.87	0.66,1.15	0.3	**Yes**
	Peri	971	0.98	0.64,1.50	>0.9	
	Rural	938	**2.38**	**1.19,4.86**	**0.015**	
**Tube well water**	Urban	813	**2.53**	**1.45,4.54**	**0.001**	**Yes**
	Peri	971	**1.37**	**1.04,1.82**	**0.027**	
	Rural	938	1.31	0.93,1.86	0.12	
**Animal owned by household**	Urban	813	**1.55**	**1.17,2.06**	**0.002**	**Yes**
	Peri	971	0.99	0.75,1.30	>0.9	
	Rural	938	**0.54**	**0.37,0.78**	**0.001**	
**Cattle or ruminant owned**	Urban	813	NA	NA	NA	No
	Peri	971	2.33	1.64,1.11	0.2	
	Rural	938	0.89	0.68,1.17	0.4	
**Poultry owned**	Urban	813	**1.61**	**1.15,2.26**	**0.005**	**Yes**
	Peri	971	0.85	0.64,1.11	0.2	
	Rural	938	0.98	0.72,1.32	0.9	
**Pet owned**	Urban	813	1.24	0.90,1.69	0.2	**Yes**
	Peri	971	0.85	0.62,1.17	0.3	
	Rural	938	**0.66**	**0.48,0.91**	**0.012**	
**Pig owned**	Urban	813	NA	NA	NA	**Yes**
	Peri	971	**0.30**	**0.09,0.78**	**0.026**	
	Rural	938	0.93	0.66,1.31	0.7	
**Animal kept inside house**	Urban	813	**1.50**	**1.05,2.15**	**0.024**	**Yes**
	Peri	971	1.26	0.94,1.67	0.12	
	Rural	938	**1.16**	**1.18,2.19**	**0.003**	
**Animal interacting with food**	Urban	813	**1.69**	**1.21,2.36**	**0.002**	**Yes**
	Peri	971	**1.38**	**1.05,1.81**	**0.020**	
	Rural	938	**1.41**	**1.07,1.85**	**0.014**	
**Animal faeces seen**	Urban	813	1.02	0.77,1.34	>0.9	No
	Peri	971	1.08	0.77,1.52	0.7	
	Rural	938	NA	NA	NA	
**River water exposure**	Urban	813	0.89	0.65,1.21	0.5	**Yes**
	Peri	971	1.33	1.00,1.78	0.054	
	Rural	938	**1.61**	**1.18,2.19**	**0.003**	
**Drain water exposure**	Urban	813	0.76	0.44,1.29	0.3	**Yes**
	Peri	971	1.30	0.86,1.94	0.2	
	Rural	938	**0.49**	**0.27,0.84**	**0.013**	
**Street food use**	Urban	813	0.51	0.33,1.09	0.2	**Yes**
	Peri	971	1.56	1.01,2.49	0.053	
	Rural	938	**1.65**	**1.19,2.29**	**0.003**	
**Shared plates**	Urban	813	0.79	0.58,1.09	0.2	**Yes**
	Peri	971	1.12	0.85,1.47	0.4	
	Rural	938	**0.71**	**0.54,0.94**	**0.016**	
**Market produce used**	Urban	813	0.65	0.40,1.05	0.08	No
	Peri	971	0.69	0.45,1.05	0.078	
	Rural	938	0.95	0.72,1.26	0.7	

S12b Table. Regional univariate analysis of WASH and individual variables against human ESBL *K. pnuemoniae* colonisation

Characteristic	Region	n	OR	95% CI	p value	Model inclusion
**Season (wet)**	Urban	813	1.30	0.85,2.00	0.2	**Yes**
	Peri	971	**1.94**	**1.27,3.02**	**0.003**	
	Rural	938	**2.19**	**1.47,3.31**	**<0.001**	
**Male sex**	Urban	813	0.69	0.43,1.09	0.12	No
	Peri	971	1.12	0.74,1.68	0.6	
	Rural	938	0.84	0.56,1.23	0.4	
**Age (log)**	Urban	813	0.97	0.82,1.17	0.8	No
	Peri	971	0.98	0.82,2.60	0.2	
	Rural	938	1.02	0.86,1.21	0.8	
**ABU** *(Last 6 months)*	Urban	813	1.28	0.74,2.11	0.4	**Yes**
	Peri	971	0.75	0.34,1.46	0.4	
	Rural	938	**1.54**	**0.99,2.35**	**0.048**	
**HIV reactive**	Urban	813	1.12	0.45,2.41	0.8	**Yes**
	Peri	971	**2.29**	**1.15,4.24**	**0.012**	
	Rural	938	0.52	0.20,1.13	0.14	
**Household density (log)**	Urban	813	**2.29**	**1.32,3.99**	**0.003**	**Yes**
	Peri	971	0.95	0.57,1.60	0.8	
	Rural	938	**2.12**	**1.24,3.60**	**0.006**	
**Income** *(>40,000MK/month)*	Urban	813	1.16	0.76,1.78	0.5	No
	Peri	971	1.10	0.73,1.67	0.7	
	Rural	938	1.04	0.71,1.54	0.8	
**Shared Toilet**	Urban	813	1.39	0.91,2.16	0.13	No
	Peri	971	0.87	0.55, 1.34	0.5	
	Rural	938	1.32	0.85,2.00	0.2	
**Drophole Present**	Urban	813	1.07	0.60,1.81	0.8	No
	Peri	971	0.96	0.61, 1.47	0.9	
	Rural	938	1.33	0.89, 1.98	0.2	
**Cleaning Materials available**	Urban	813	1.07	0.64,1.73	0.8	No
	Peri	971	1.06	0.70,1.59	0.8	
	Rural	938	0.78	0.39,1.45	0.5	
**Human Feaces visible**	Urban	813	**1.58**	**1.03,2.44**	**0.039**	**Yes**
	Peri	971	1.44	0.90,2.25	0.12	
	Rural	938	1.01	0.68,1.49	>0.9	
**Human defecation practiced**	Urban	813	0.79	0.19,2.30	0.7	No
	Peri	971	0.75	0.36,1.41	0.4	
	Rural	938	0.64	0.31,1.21	0.2	
**HWF present**	Urban	813	1.55	1.00,2.42	0.053	No
	Peri	971	1.02	0.60,1.86	>0.9	
	Rural	938	1.29	0.88,1.93	0.2	
**Soap present**	Urban	813	1.19	0.56,2.30	0.6	No
	Peri	971	0.72	0.46,1.10	0.13	
	Rural	938	0.31	0.05,1.04	0.11	
**Stored water covered**	Urban	813	0.83	0.46,1.58	0.5	No
	Peri	971	0.73	0.48,1.12	0.14	
	Rural	938	1.08	0.73,1.60	0.7	
**Stored water covered and tap**	Urban	813	0.84	0.54,1.31	0.5	No
	Peri	971	0.68	0.28,1.42	0.3	
	Rural	938	1.06	0.71,1.58	0.8	
**Utensil water**	Urban	813	0.80	0.50,1.26	0.3	No
	Peri	971	0.62	0.25,1.28	0.2	
	Rural	938	0.88	0.49,1.48	0.6	

**Piped water (i.e. kiosk)**	Urban	813	1.53	1.00,2.34	0.052	No
	Peri	971	0.85	0.52,1.36	0.5	
	Rural	938	1.36	0.84,2.15	0.2	
**Tap water (i.e. household tap)**	Urban	813	0.66	0.43,1.02	0.061	No
	Peri	971	1.31	0.69,2.33	0.4	
	Rural	938	0.21	0.01, 1.00	0.13	
**Tube well water**	Urban	813	0.91	0.34,2.03	0.8	No
	Peri	971	1.08	0.71,1.67	0.7	
	Rural	938	0.80	0.51, 1.28	0.3	
**Animal owned by household**	Urban	813	1.39	0.91,2.13	0.13	No
	Peri	971	0.81	0.53,1.22	0.3	
	Rural	938	0.70	0.43, 1.20	0.2	
**Cattle or ruminant owned**	Urban	813	NA	NA	NA	No
	Peri	971	1.06	0.59,1.80	0.8	
	Rural	938	1.22	0.83,1.80	0.3	
**Poultry owned**	Urban	813	**1.46**	**1.12,2.87**	**0.013**	**Yes**
	Peri	971	1.12	0.74,1.69	0.6	
	Rural	938	1.37	0.88,2.21	0.2	
**Pet owned**	Urban	813	1.33	0.55,1.47	0.7	**Yes**
	Peri	971	1.01	0.61,1.62	>0.9	
	Rural	938	**1.59**	**1.05,2.39**	**0.027**	
**Pig owned**	Urban	813	NA	NA	NA	No
	Peri	971	NA	NA	NA	
	Rural	938	1.56	0.98,2.42	0.055	
**Animal kept inside house**	Urban	813	1.46	0.87,2.40	0.14	**Yes**
	Peri	971	0.52	0.30,0.86	**0.014**	
	Rural	938	**1.48**	**1.00,2.19**	**0.048**	
**Animal interacting with food**	Urban	813	1.33	0.80,2.15	0.3	No
	Peri	971	0.92	0.60,1.40	0.7	
	Rural	938	0.69	0.46,1.01	0.058	
**Animal faeces seen**	Urban	813	**0.63**	**0.40,0.96**	**0.036**	**Yes**
	Peri	971	1.42	0.82,2.60	0.2	
	Rural	938	NA	NA	NA	
**River water exposure**	Urban	813	1.48	0.94,2.30	0.088	No
	Peri	971	1.28	0.82,2.04	0.3	
	Rural	938	0.81	0.49,1.28	>0.9	
**Drain water exposure**	Urban	813	1.46	0.68,2.85	0.3	**Yes**
	Peri	971	0.91	0.45,1.68	0.8	
	Rural	938	**2.82**	**1.59,4.83**	**<0.001**	
**Street food use**	Urban	813	0.52	0.30,0.92	**0.036**	**Yes**
	Peri	971	0.86	0.48,1.67	0.6	
	Rural	938	0.90	0.59,1.41	0.6	
**Shared plates**	Urban	813	0.71	0.42,1.16	0.2	**Yes**
	Peri	971	0.84	0.54,1.27	0.4	
	Rural	938	**0.64**	**0.43,0.95**	**0.025**	
**Market produce used**	Urban	813	1.13	0.56,2.63	0.7	No
	Peri	971	0.80	0.44,1.55	0.5	
	Rural	938	1.13	0.75,1.71	0.6	

S13a Table. Odds ratios for covariates in ESBL *E. coli* model.

Covariate	aOR (95% CrI)
Age	1.14 (1.05-1.25)
AnimalFoodInteraction	1.62 (1.17-2.28)
AnimalKeptInside	1.58 (1.00-2.43)
AnimalPet	0.94 (0.62-1.39)
AnimalPoultry	0.68 (0.42-1.12)
AnyAnimalOwned	1.03 (0.61-1.70)
CleaningMaterialsAny	0.80 (0.55-1.15)
CoveredStoredWater	1.00 (0.70-1.40)
DrainWaterExp	0.97 (0.56-1.64)
DropholePresent	0.82 (0.59-1.14)
HouseholdDensity	1.01 (0.68-1.47)
HumanDefecation	0.85 (0.48-1.50)
HumanFeaces	0.97 (0.70-1.32)
PipedWater	1.04 (0.46-2.27)
RegionRural	1.15 (0.72-1.83)
RegionUrban	2.01 (1.26-3.24)
RiverWaterExp	1.08 (0.78-1.51)
SeasonWet	1.66 (1.38-2.00)
SharedPlates	0.74 (0.53-1.03)
SharedToilet	1.18 (0.86-1.63)
SoapPresent	1.00 (0.66-1.55)
StreetFood	1.04 (0.66-1.63)
TapWater	1.09 (0.50-2.29)
Tubewell	1.37 (0.64-2.91)

S13b Table. Odds ratios for covariates in ESBL *K. pnuemoniae* model.

Covariate	aOR (95% CrI)
ABU	1.24 (0.87-1.74)
AnimalFaeces	0.76 (0.45-1.27)
AnimalKeptInside	0.80 (0.48-1.39)
AnimalPet	1.05 (0.69-1.57)
AnimalPoultry	1.50 (0.86-2.54)
DrainWaterExp	1.75 (0.93-3.24)
HIV	1.32 (0.79-2.22)
HouseholdDensity	1.49 (0.95-2.44)
HumanFeaces	1.43 (0.98-2.11)
RegionRural	1.24 (0.76-2.05)

RegionUrban	1.12 (0.70-1.83)
SeasonWet	2.12 (1.63-2.76)
SharedPlates	0.70 (0.47-1.04)
StreetFood	0.89 (0.54-1.49)

S14a Table. Table of parameter testing for regional adjustment of variables included in the ESBL *E. coli* mixed effects model

Characteristic	Likelihood ratio test	Adjust for Region*
**Season**	$\chi2$ (2)= 8.33, p = 0.0155	**Yes**
**Male**	NA	
**Age**	$\chi2$ (2)= 0.80, p =0.67	No
**ABU**	NA	
**HIV reactive**	NA	
**Household density**	$\chi2$ (2)= 4.82, p =0.090	No
**Income >40,000MK/month**	NA	
**Shared Toilet**	$\chi2$ (2)= 4.93, p = 0.085	No
**Drophole Present**	$\chi2$ (2)= 8.51, p = 0.014	**Yes**
**Cleaning Materials available**	$\chi2$ (2)= 7.66, p = 0.022	**Yes**
**Human Feaces visible**	$\chi2$ (2)= 7.88, p = 0.019	**Yes**
**Human defecation practiced**	$\chi2$ (2)= 5.64, p = 0.059	No
**HWF present**	NA	
**Soap present**	$\chi2$ (2)= 14.32, p = <0.001	**Yes**
**Stored water covered**	$\chi2$ (2)= 26.52, p = <0.001	**Yes**
**Stored water covered and tap**	NA	
**Utensil water**	NA	
**Piped water (i.e. kiosk)**	$\chi2$ (2)= 4.39, p =0.111	No
**Tap water (i.e. household tap)**	$\chi2$ (2)= 6.92, p =0.031	**Yes**
**Tube well water**	$\chi2$ (2)= 4.28, p =0.117	No
**Animal owned by household**	$\chi2$ (2)= 19.61, p = <0.001	**Yes**
**Cattle or ruminant owned**	NA	
**Poultry owned**	$\chi2$ (2)= 8.91, p =0.011	**Yes**
**Pet owned**	$\chi2$ (2)= 7.63, p =0.022	**Yes**
**Pig owned**	NA	
**Animal kept inside house**	$\chi2$ (2)= 1.34, p = 0.510	No
**Animal interacting with food**	$\chi2$ (2)= 0.94, p = 0.624	No
**Animal faeces seen**	NA	
**River water exposure**	$\chi2$ (2)= 7.36, p = 0.025	**Yes**
**Drain water exposure**	$\chi2$ (2)= 8.23, p = 0.016	**Yes**
**Street food use**	$\chi2$ (2)= 20.84, p = <0.001	**Yes**
**Shared plates**	$\chi2$ (2)= 5.72, p = 0.057	No
**Market produce used**	NA	

**An alpha level 0.05 has been used as a cut off for the decision to adjust for regional effects in the final mixed effect model.*

S14b Table. Table of parameter testing for regional adjustment of variables included in the ESBL *K. pnuemoniae* mixed effects model

Variable	Likelihood ratio test	Adjust for Region*
**Season**	$\chi 2$ (2)= 3.25, p=0.197	No
**Male**	NA	
**Age**	NA	No
**ABU**	$\chi 2$ (2)= 3.08, p=0.215	No
**HIV reactive**	$\chi 2$ (2)= 7.87, p=0.020	**Yes**
**Household density**	$\chi 2$ (2)= 6.53 , p= 0.038	**Yes**
**Income >40,000MK/month**	NA	
**Shared Toilet**	NA	
**Drophole Present**	NA	
**Cleaning Materials available**	NA	
**Human Feaces visible**	$\chi 2$ (2)= 2.56, p=0.278	No
**Human defecation practiced**	NA	
**HWF present**	NA	
**Soap present**	NA	
**Stored water covered**	NA	
**Stored water covered and tap**	NA	
**Utensil water**	NA	
**Piped water (i.e. kiosk)**	NA	
**Tap water (i.e. household tap)**	NA	
**Tube well water**	NA	
**Animal owned by household**	NA	
**Cattle or ruminant owned**	NA	
**Poultry owned**	$\chi 2$ (2)= 2.24, p=0.327	No
**Pet owned**	$\chi 2$ (2)= 3.46, p=0.177	No
**Pig owned**	NA	
**Animal kept inside house**	$\chi 2$ (2)= 12.39, p=0.002	**Yes**
**Animal interacting with food**	NA	
**Animal faeces seen**	$\chi 2$ (2)= 5.56, p=0.062	No
**River water exposure**	NA	
**Drain water exposure**	$\chi 2$ (2)= 7.02, p=0.030	**Yes**
**Street food use**	$\chi 2$ (2)= 2.54, p=0.281	No
**Shared plates**	$\chi 2$ (2)= 0.81, p=0.665	No
**Market produce used**	NA	

**An alpha level 0.05 has been used as a cut off for the decision to adjust for regional effects in the final mixed effect model.*

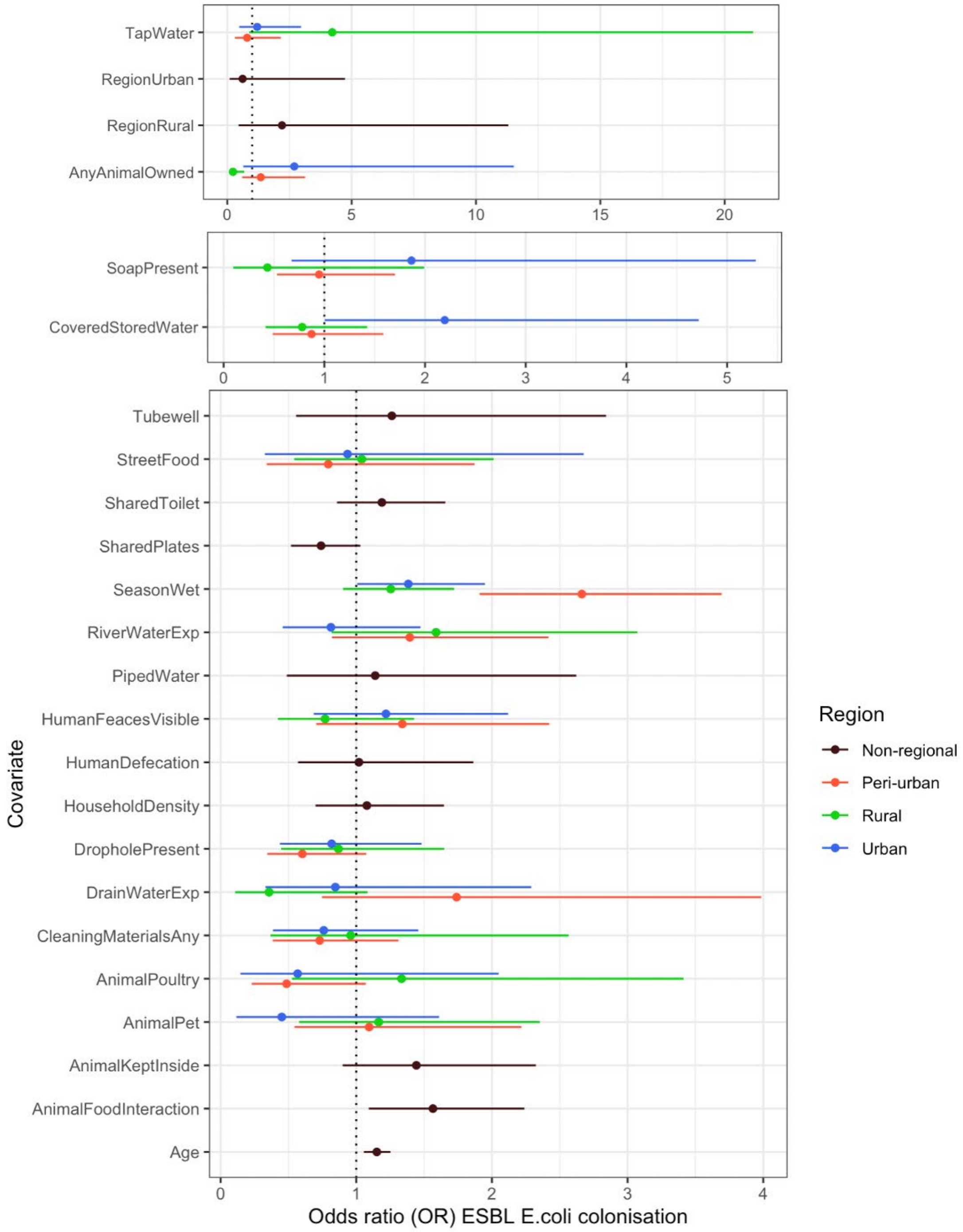

S1a Fig. Parameter estimates for the fixed-effects used in a multivariable model of ESBL *E. coli* colonisation, expressed as odds ratios with 95% CrI. Covariates either included an interaction term by region (and so their effect varies by region - red=peri-urban, green=rural or blue=urban) or had the same effect across region (black). **Covariates that were significantly associated (p<0.05) with colonisation via univariable analysis in any region were evaluated for a different effect across regions by comparing models with and without a covariate*region interaction term using likelihood ratio testing for both ESBL E. coli and ESBL K. pneumoniae. An interaction term with region was included for those covariates for which p < 0.05 on likelihood ratio testing **(S12a Table)**.*

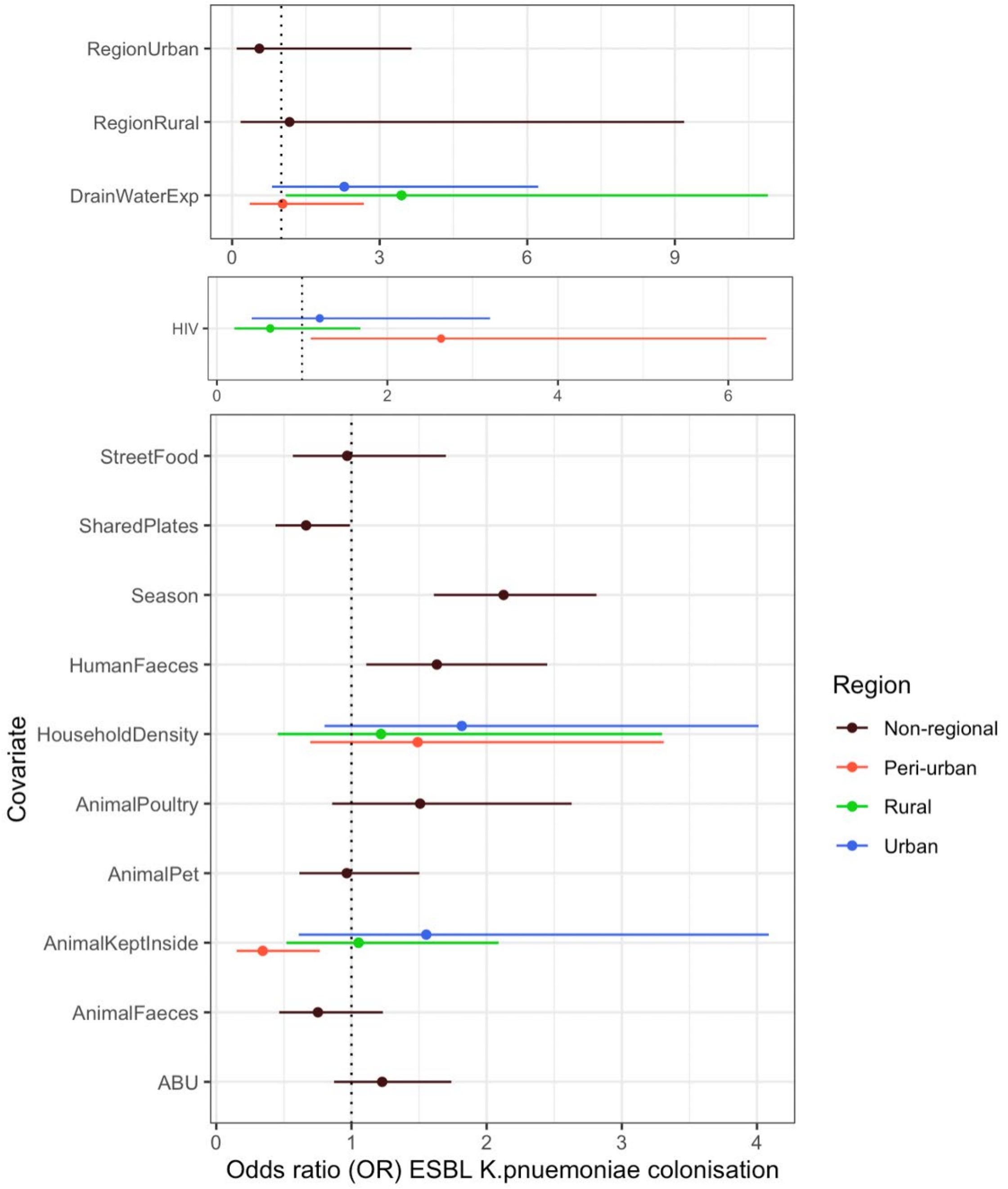

S1b Fig. Parameter estimates for the fixed-effects used in a multivariable model of ESBL *K. pneumoniae* colonisation, expressed as odds ratios with 95% CrI. Covariates either included an interaction term by region (and so their effect varies by region - red=peri-urban, green=rural or blue=urban) or had the same effect across regions (black). **Covariates that were significantly associated (p<0.05) with colonisation via univariable analysis in any region were evaluated for a different effect across regions by comparing models with and without a covariate*region interaction term using likelihood ratio testing for both ESBL E. coli and ESBL K. pneumoniae. An interaction term with region was included for those covariates for which p < 0.05 on likelihood ratio testing* ***(S12b Table)***.

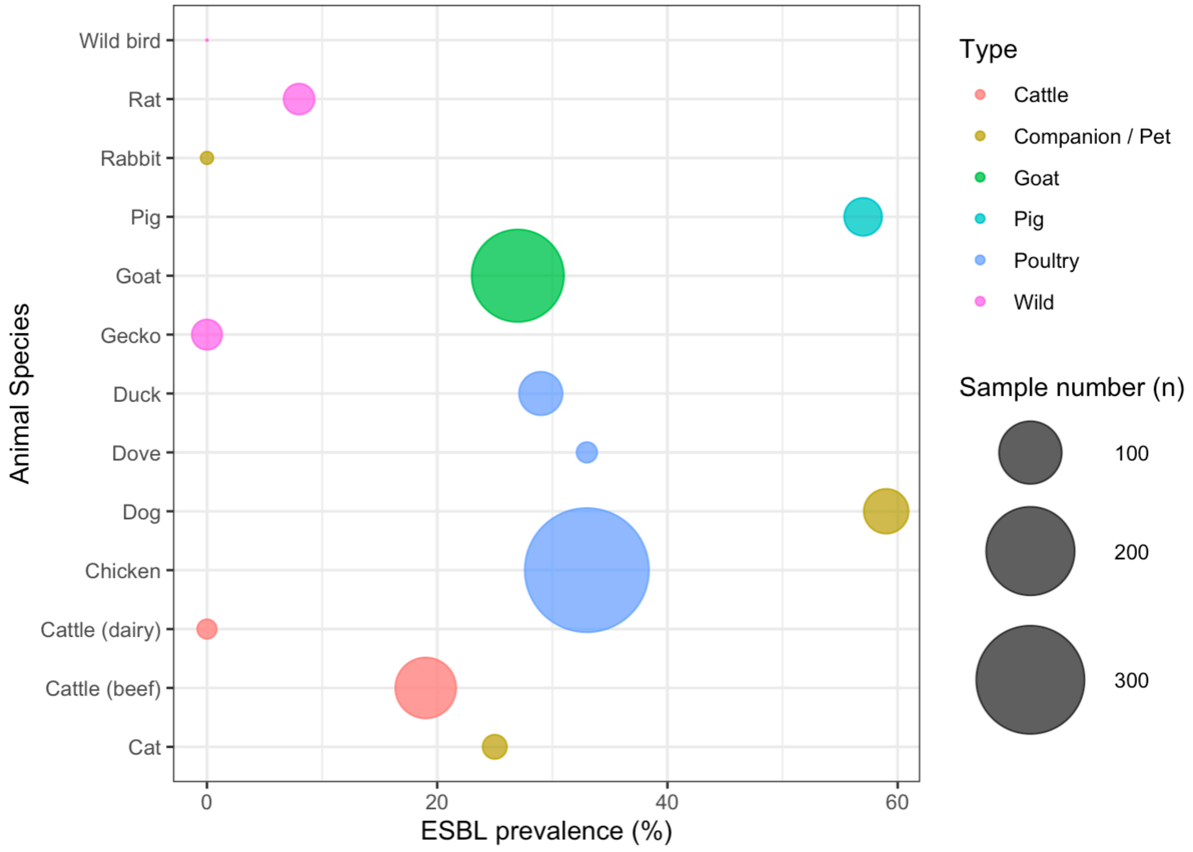

S2 Fig. Bubble plot of ESBL-E (*E. coli* or *K. pneumoniae*) prevalence in animal stool samples, stratified by species, and coloured by animal type. The volume of the circle represents the number of samples processed for each species.

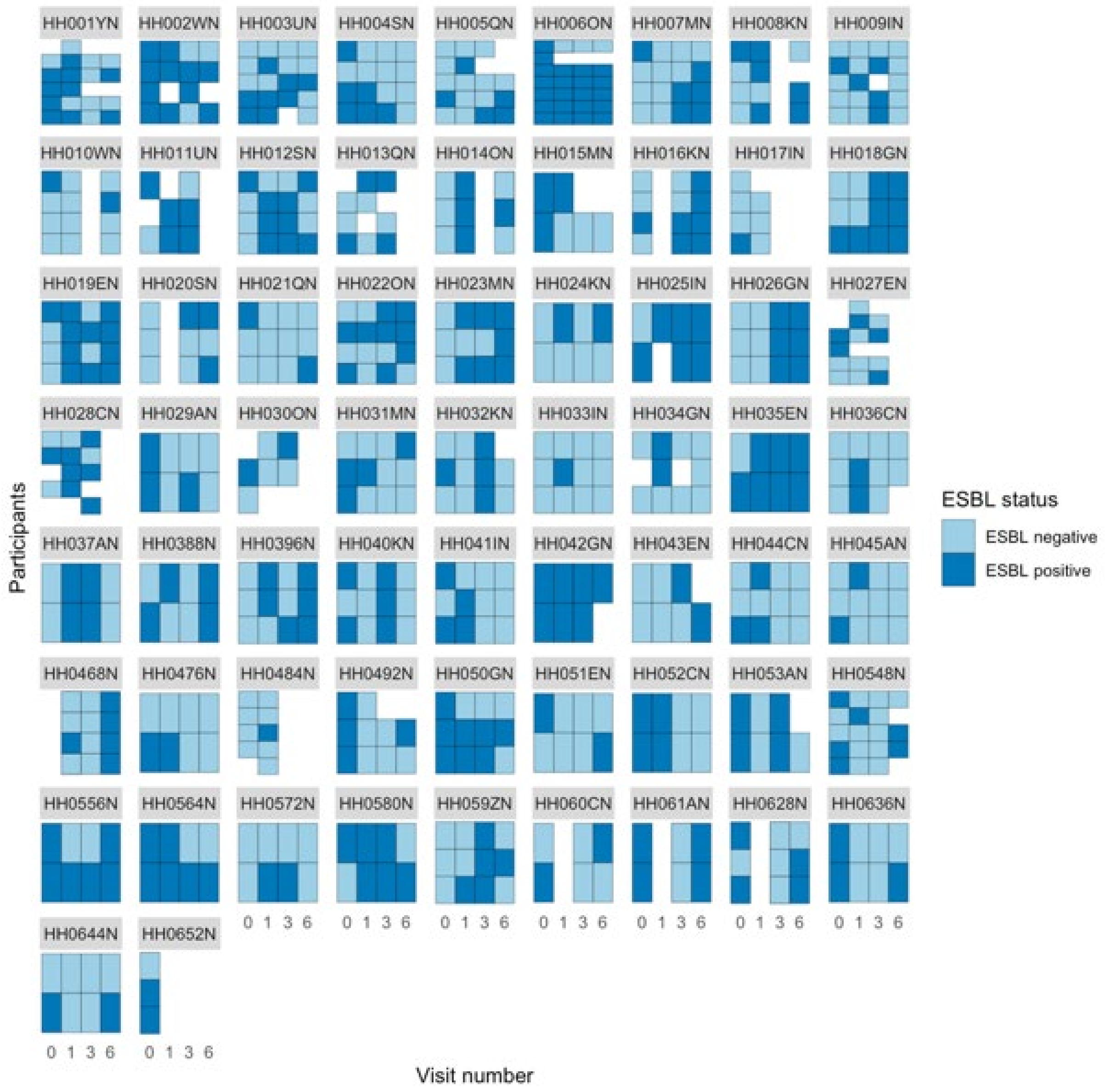

S3a Fig. Facet Plot showing flux of human ESBL (*E. coli* or *K. pneumoniae*) colonisation amongst **urban** household members over time, grouped by the 65 households recruited. Each row represents a participant, each column represents a visit, and each small square is a sample coloured by EBSL status (positive or negative). Where no sample was returned for an individual at a visit the square remains blank.

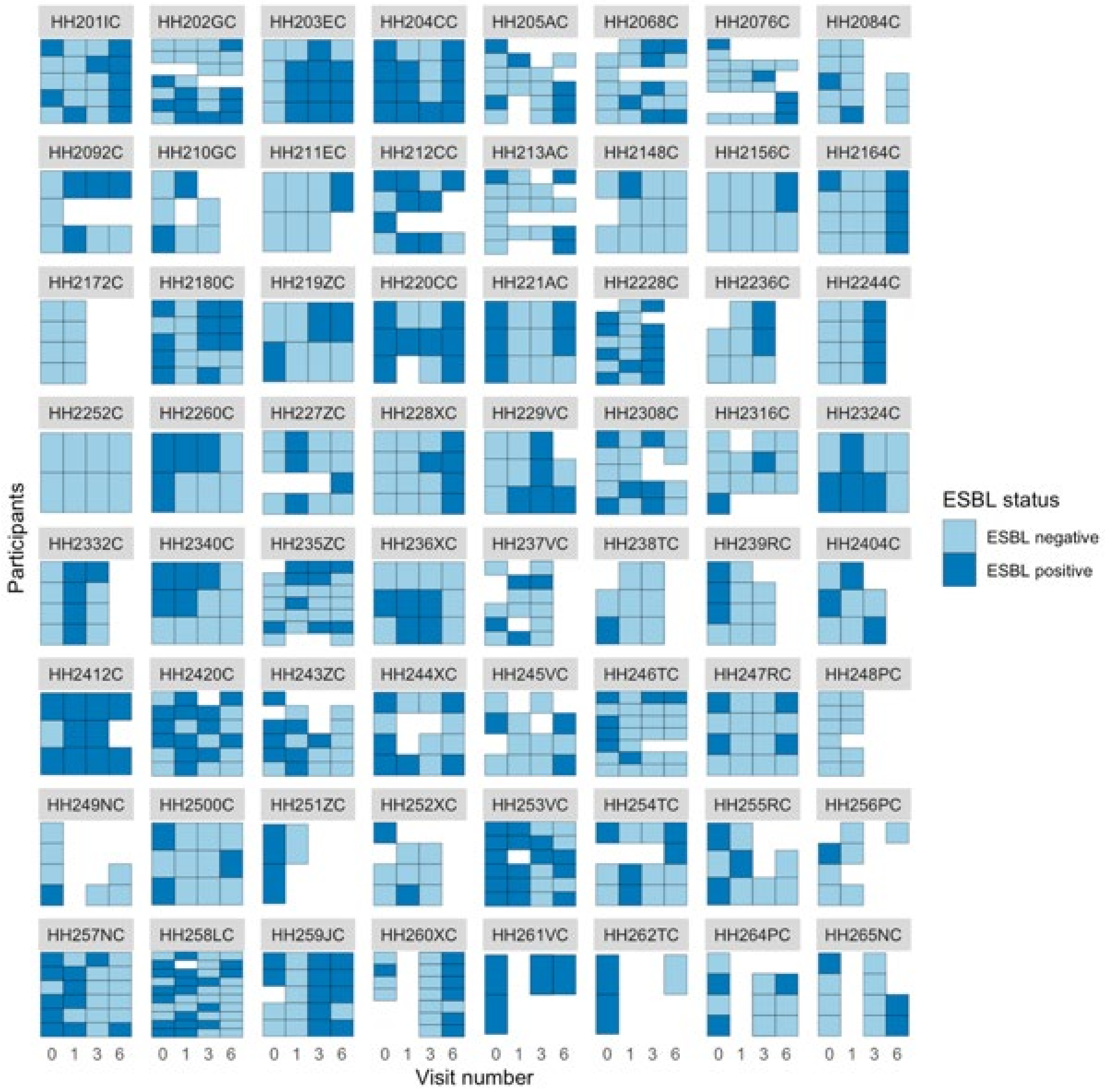

S3b Fig. Facet Plot showing flux of human ESBL colonisation (*E. coli* or *K. pneumoniae*) amongst **peri-urban** household members over time, grouped by the 65 households recruited. Each row represents a participant, each column represents a visit, and each small square is a sample coloured by EBSL status (positive or negative). Where no sample was returned for an individual at a visit the square remains blank.

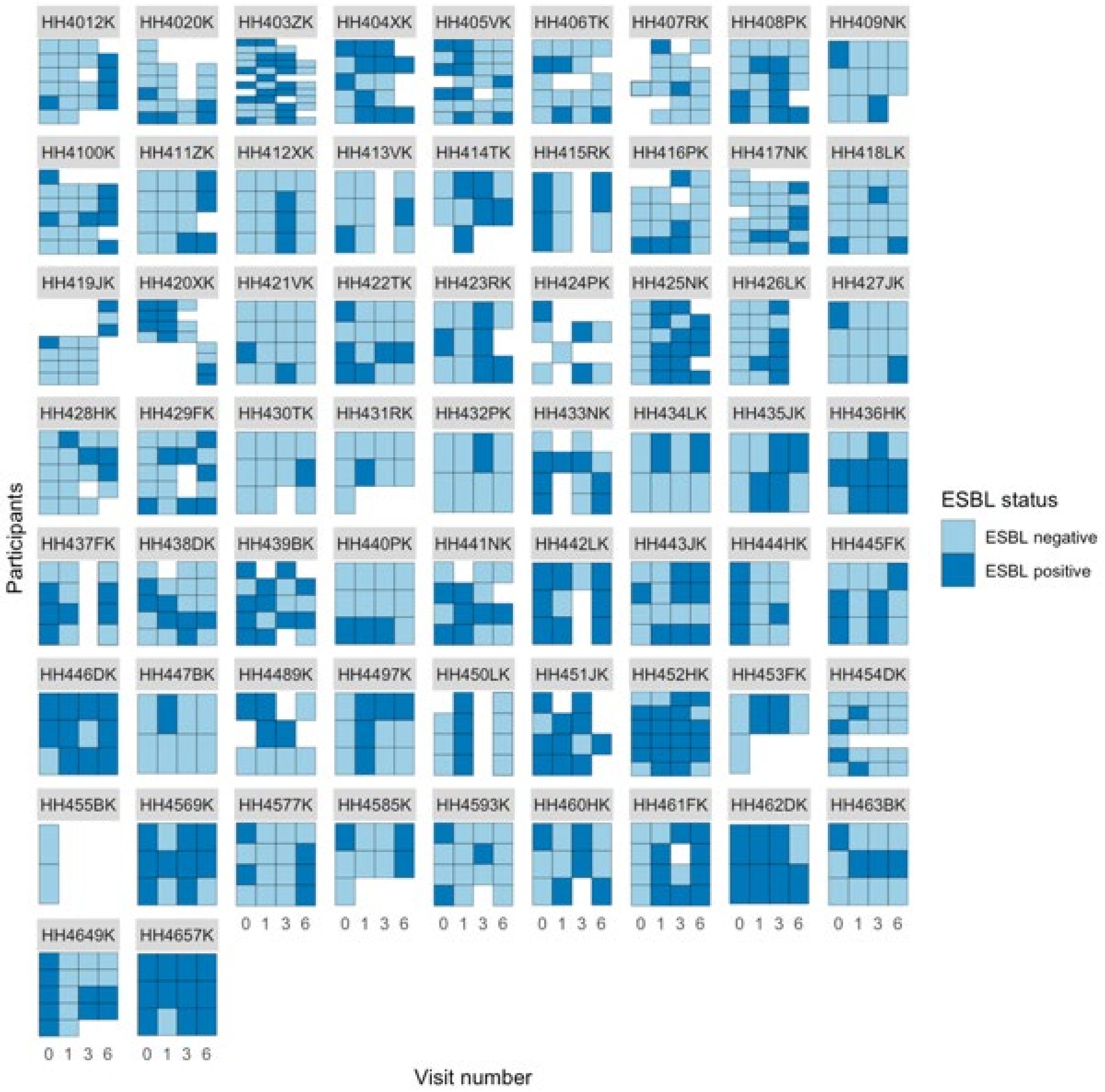

S3c Fig. Facet Plot showing flux of human ESBL (*E. coli* or *K. pneumoniae*) colonisation amongst **rural** household members over time, grouped by the 65 households recruited. Each row represents a participant, each column represents a visit, and each small square is a sample coloured by EBSL status (positive or negative). Where no sample was returned for an individual at a visit the square remains blank.

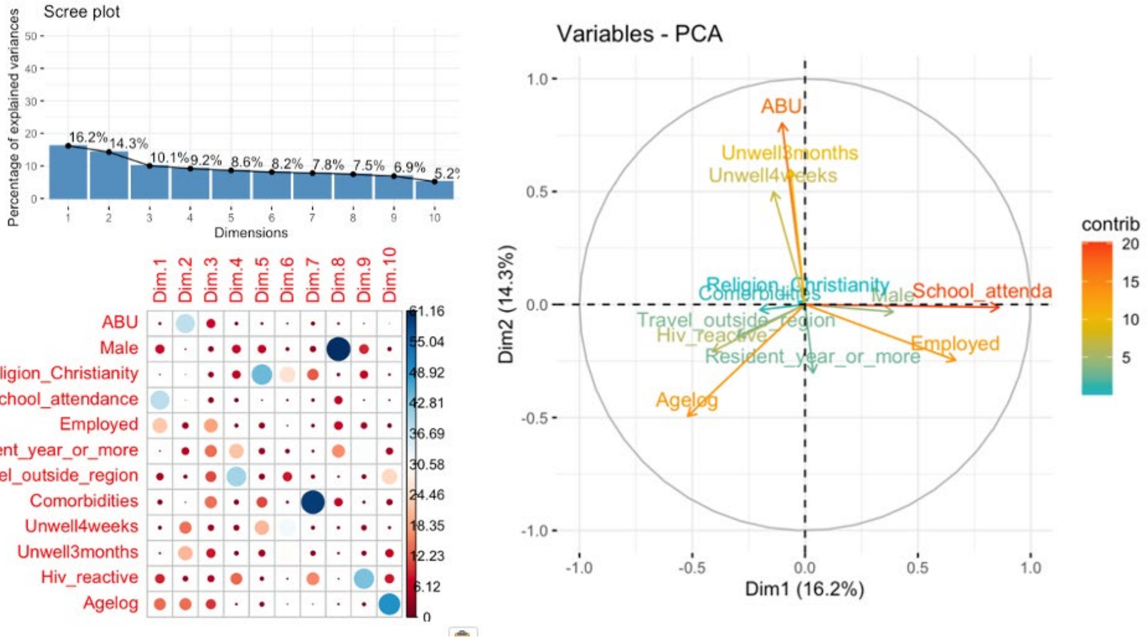

S4a Fig. PCA analysis of individual variables, including a scree plot of the eigenvalues (top left), weighting of the variables by PCA (bottom left), and factor map (right).

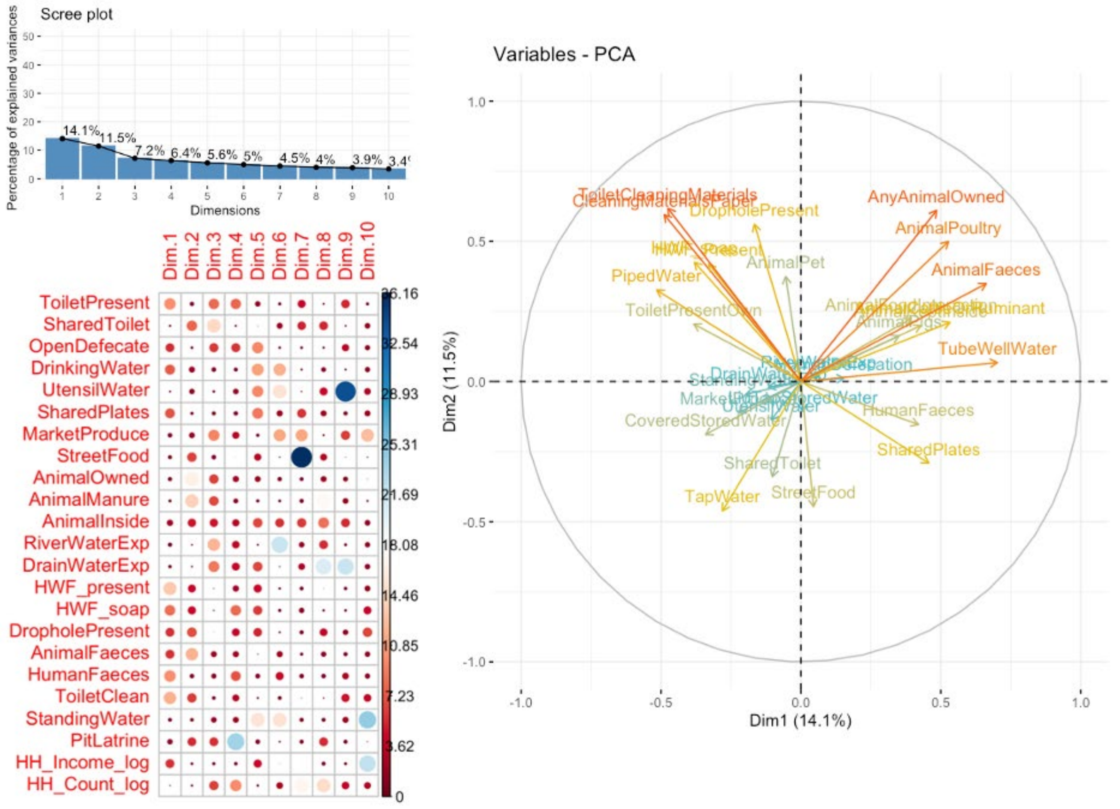

S4b Fig. PCA analysis of household variables, including a scree plot of the eigenvalues (top left), weighting of the variables by PCA (bottom left), and factor map (right).

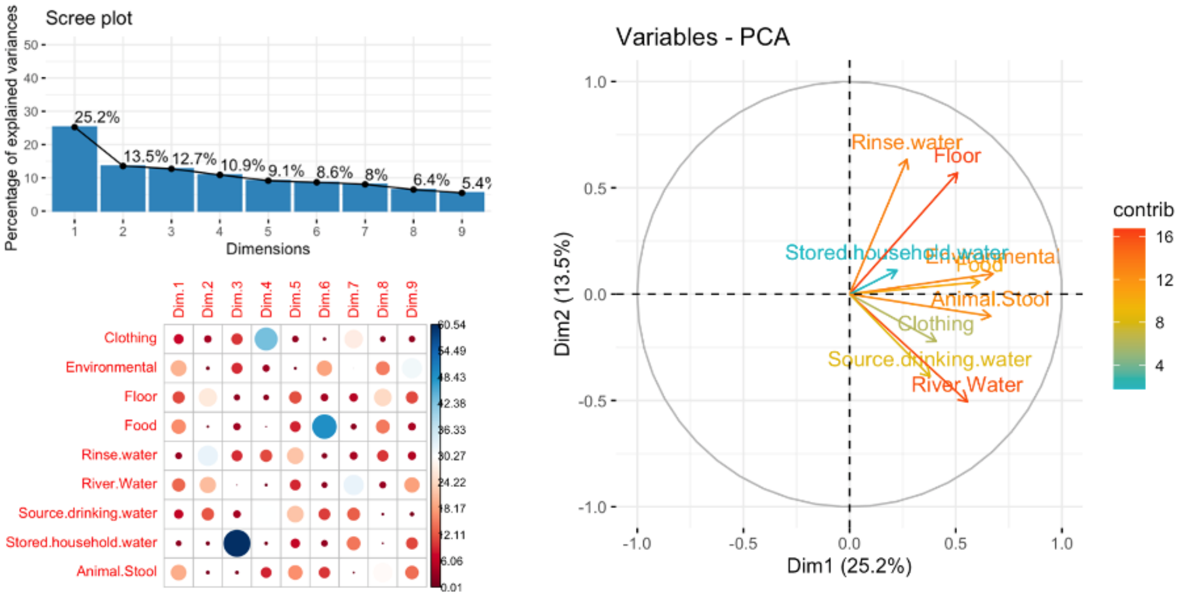

S4c Fig. PCA analysis of environmental contamination variables, including a scree plot of the eigenvalues (top left), weighting of the variables by PCA (bottom left), and factor map (right).

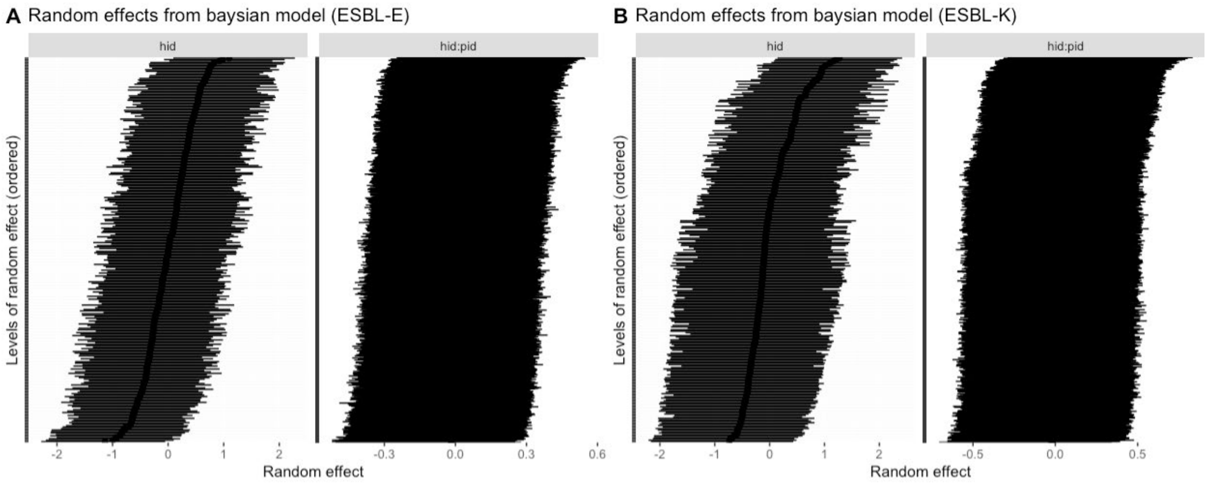

S5 Fig. Random effects from Bayesian multivariate models of (a) ESBL *E. coli* [ESBL-E], and (b) ESBL *K. pnuemoniae* [ESBL-K], inclusive of within household (hid) and within participant (hid:pid) effects.
